# Conditions for establishing the “generalized Snell’s law of refraction” in all-dielectric metasurfaces: theoretical bases for design of high-efficiency beam deflection metasurfaces

**DOI:** 10.1515/nanoph-2021-0459

**Published:** 2021-11-01

**Authors:** Siyuan Shen, Zhaohui Ruan, Yuan Yuan, Heping Tan

**Affiliations:** School of Energy Science and Engineering, Harbin Institute of Technology, 92 West Dazhi Street, Harbin 150001, P. R. China; Key Laboratory of Aerospace Thermophysics, Ministry of Industry and Information Technology, Harbin Institute of Technology, 92 West Dazhi Street, Harbin 150001, P. R. China

**Keywords:** anomalous refraction, deflection efficiency, generalized Snell’s law, metasurfaces, nanophotonics

## Abstract

The generalized Snell’s law dictates that introducing a phase gradient at the interface of two media can shape incident light and achieve anomalous reflection or refraction. However, when the introduced phase gradient is realized via the scattering of nanoparticles in the metasurfaces, this law needs to be modified; certain conditions need to be met when the law is established. We present the conditions for establishing the “generalized Snell’s law of refraction” in all-dielectric metasurfaces under the incidence of different polarized light. These conditions can provide theoretical bases for the subsequent design of high-efficiency beam deflection metasurfaces. The relationship between the highest achievable anomalous refraction efficiency and the number of nanoparticles within one period of the metasurface is also summarized. In addition, the generalized refraction should not depend on the polarization states of incident light; however, the previous realization conditions of anomalous refraction were sensitive to the polarization states. Thus, conditions for establishing the polarization-independent generalized Snell’s law of refraction in all-dielectric metasurfaces are presented.

## 1 Introduction

Conventional optical devices rely on the phase shifts accumulated during the propagation of light to shape light beams [1]. However, the phase discontinuities introduced by two-dimensional (2D) nanoparticle arrays (metasurfaces) disrupt the dependence of light on the phase accumulation during propagation; this also enables the metasurfaces to locally modify the phase, polarization state, and intensity of light beams at the subwavelength scale [2]. Currently, it is possible to manufacture metasurfaces with nanometer-scale precision and a wide range of physical parameters and materials, which provides almost any amount of freedom for the design of new optical components [3], [4], [5]. Among these, all-dielectric metasurfaces have attracted extensive attention owing to their high efficiency and low loss [6].

A basic function of metasurfaces is the realization of light beam deflection (i.e., to reflect or refract incident light to the desired direction). Over recent years, some previous studies have studied the conditions required for metasurfaces to realize efficient light beam deflection. For instance, in 2011, Yu et al. [1] proposed generalized laws of reflection and refraction and showed that anomalous reflection can be observed when metallic antennas on silicon within one period of the metasurface array have a linear phase change along the interface. In 2020, Rousseau et al. [7] reported that the above-mentioned generalized laws of refraction and reflection have to be modified to describe the propagation of light through metasurfaces composed of diffractive elements. They also theoretically proved that generalized reflection or refraction can only be achieved when nanoparticles within one period of the metasurface introduce a phase jump with a slope of 2*π*/(the metasurface period). At this time, the form of the generalized laws of reflection and refraction are completely transformed into the grating diffraction equations. However, the conditions to achieve generalized reflection or refraction, as stated in the above literature, remain unclear. Furthermore, the research on designing metasurfaces based on the generalized Snell’s law has not yielded a specific rule for designing efficient beam deflection metasurfaces. For example, previous studies [1, 8], [9], [10], [11], [12], [13], [14], [15], [16], [17], [18] have indicated that the nanoparticles within one period of the metasurface array must introduce a phase gradient with a linear distribution in the 2*π* range in order to achieve anomalous refraction or reflection. However, the phase gradients along the interface introduced by the actual selected nanoparticles failed to reach 2*π* in literature [8], [9], [10], [11], [12], [13], [14], [15], [16], [17]. The phase gradients in literature [18] reached such a range; however, the efficiency of anomalous refraction they realized was only greater than 60%. Therefore, according to the above-discussed literature and analysis, the conditions that metasurfaces need to meet in order to achieve generalized reflection or refraction have not been unified nor clarified thus far. It is, therefore, necessary to further explore and analyze the specific principles for designing efficient beam deflection metasurfaces.

For the development and design of beam steering metasurfaces, it is also necessary to understand the maximum achievable value of anomalous refraction (reflection) efficiency of the metasurfaces, which can be expressed as transmission (reflection) efficiency in the anomalous refraction (reflection) direction/total transmission (reflection) efficiency. However, few studies have focused on the efficiency of anomalous reflection or refraction achieved by metasurfaces. In 1989, a diffraction efficiency formula for multi-level phase gratings was proposed [19]; it served as a basis for subsequent research on the anomalous reflection or refraction efficiency. Subsequently, in 2021, Isić et al. [5] formulated a theoretical model for the beam deflection efficiency in reflective metasurfaces, based on the scalar diffraction theory [20], Fourier optics theory [21], and temporal coupled-mode theory [22]. To the best of our knowledge, none of the previous studies have focused on the efficiency of beam deflection in transmissive metasurfaces.

Based on the background, this study aims to further study and analyze the establishment conditions for the generalized Snell’s law of refraction in transmissive metasurfaces under the incidence of different polarized light, to provide specific rules for designing efficient beam deflection metasurfaces and to give the highest achievable efficiency value of anomalous refraction. We emphasize that we are currently only concerned with metasurfaces composed by the unit cell containing one nanoparticle, and this study assumes that the interaction between the nanoparticles and light is limited within the particles and is not affected by the scattering of the surrounding particles [1, 2].

## 2 Theory

**Figure 1:**
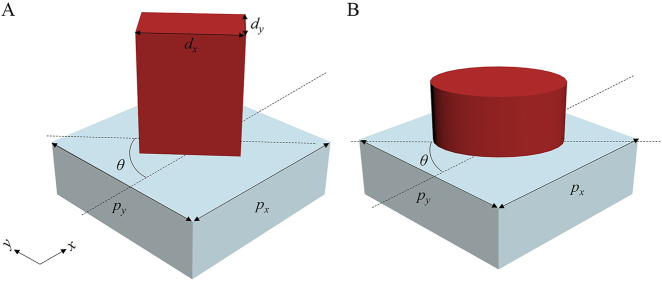
Dielectric nanopillar unit structure. (A) Rectangular. (B) Elliptical nanopillars.

The metasurfaces explored in this study are composed of all-dielectric nanoparticles, whose Jones matrix acts as a linear birefringent, unitary matrix. Over recent years, this type of metasurfaces has emerged as the most widely studied and most common all-dielectric metasurfaces. They can be realized using rectangular or elliptical dielectric nanopillars that exhibit birefringence (Figure 1). The transverse section of the dielectric nanopillars features two perpendicular symmetry axes; therefore, two modes related to the polarization states of incident light are expected to propagate with different indices in the pillars. Thus, the pillars can behave as wave plates [23]. For the pillars in Figure 1, the Jones matrix can be expressed as [2, 24], [25], [26], [27].

(1) $$J=R\left(-\theta \right)[\begin{array}{l} e^{i\phi_{x}}\\ 0 \end{array}\begin{array}{r} 0\\ e^{i\phi_{y}} \end{array}]R\left(\theta \right).$$

Here, *R*(*θ*) is a 2 × 2 rotation matrix, and *θ* denotes the rotation angle of the pillar element (Figure 1). *ϕ*_*x*_ represents the phase change introduced by the nanopillars to the *x*-polarized (0° linear polarized) incident light, and *ϕ*_*y*_ shows the phase change introduced by the nanopillars to the *y*-polarized (90° linear polarized) incident light. Either these phase changes can be independently controlled by designing the geometric dimensions of the nanopillars (the phase change introduced using this method is termed as the propagation phase) or by adjusting the rotation angles of the nanopillars (the phase change introduced using this method is termed as the geometric phase). Eq. (1) implies that the nanopillars only regulate the phase of incident light but not the amplitude. It also implies two restrictions [23]: the Jones matrix must be unitary and linearly birefringent.

**Figure 2:**
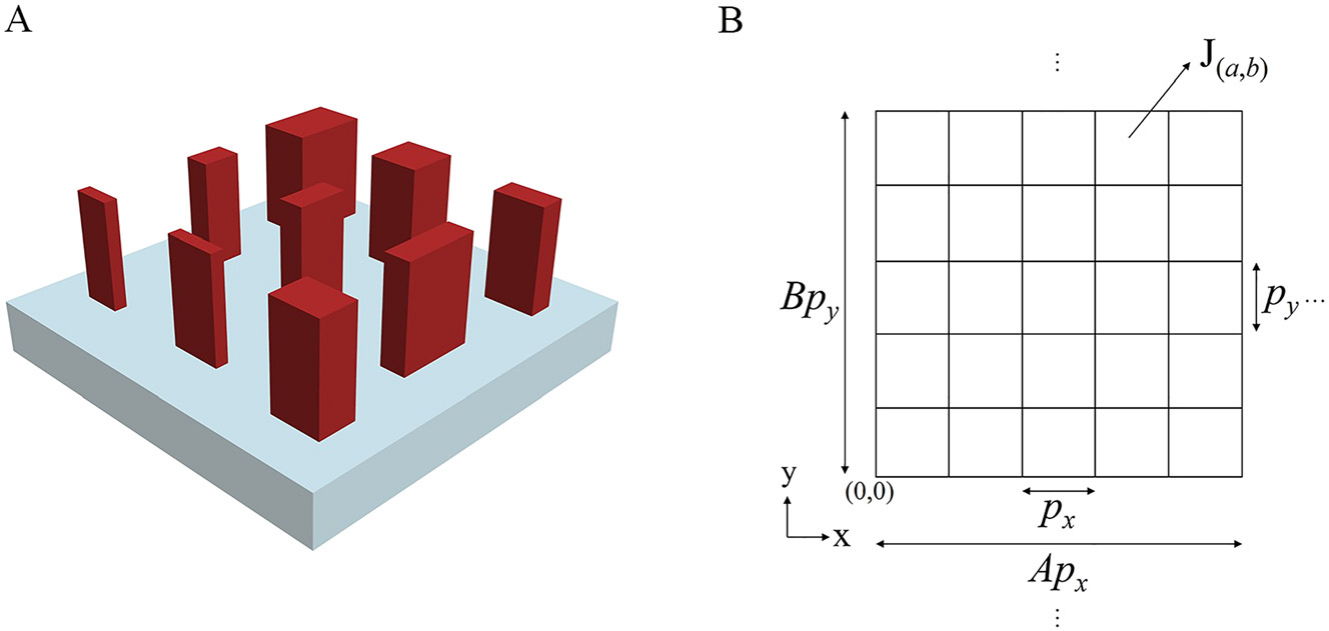
Basic structure of supercells of the beam deflection metasurfaces and simplified representation of the 2D grid of the metasurface grating structure. (A) Basic structure of the supercells. (B) Simplified representation of the 2D grid of the metasurface grating.

The beam deflection metasurfaces studied are composed of periodic supercells. The basic structure of the supercell is shown in Figure 2A. Each supercell is composed of *A* × *B* dielectric nanopillar units. Each nanopillar unit, *l*, can introduce a phase variation, *ϕ*_*xl*_, to the *x*-polarized incident light and a phase variation, *ϕ*_*yl*_, to the *y*-polarized incident light. The periods of each unit are *p*_*x*_ along the *x*-direction and *p*_*y*_ along the *y*-direction. As the metasurfaces are periodic arrays, each item in the Jones matrix of the nanopillars should also be periodic. In other words, for any nanopillar *l*, *ϕ*_*x*(*l* + *A*)_ = *ϕ*_*xl*_, *ϕ*_*y*(*l* + *B*)_ = *ϕ*_*yl*_. Figure 2B simplifies the metasurface grating structure with a 2D grid. The position of the origin is depicted in Figure 2B. Each grid represents a nanopillar, and the position is represented by (*a*, *b*), where *a*, *b* are integers and 1≤a≤A,1≤b≤B . Therefore, the Jones matrix of the nanopillar at the position (*a*, *b*) can be expressed as [23].

(2) $$J_{\left(a,b\right)}=R\left(-\theta_{\left(a,b\right)}\right)[\begin{array}{l} e^{i\phi_{x\left(a,b\right)}}\\ 0 \end{array}\begin{array}{r} 0\\ e^{i\phi_{y\left(a,b\right)}} \end{array}]R\left(\theta_{\left(a,b\right)}\right).$$

Here, *θ*_(*a*,*b*)_, *ϕ*_*x*(*a*,*b*)_, and *ϕ*_*y*(*a*,*b*)_ represent the rotation angle of the nanopillar, phase variation introduced to the *x*-polarized incident light, and phase variation introduced to the *y*-polarized incident light at the position (*a*, *b*), respectively.

According to Eq. (2), we can obtain the expression of the Jones matrix at each position of the metasurface:

(3) $$J_{\left(x,y\right)}=\left(\sum_{a=1}^{A}\sum_{b=1}^{B}J_{\left(a,b\right)}{\text{rect}}_{p_{x},p_{y}}\left(x-\left(a-1/2\right)\cdot p_{x},y-\left(b-1/2\right)\cdot p_{y}\right)\right)⊗{\text{comb}}_{p_{x},p_{y}}\left(x,y\right).$$

Here, ${\text{rect}}_{p_{x},p_{y}}\left(x-\left(a-1/2\right)\cdot p_{x},y-\left(b-1/2\right)\cdot p_{y}\right)=\left\{ \begin{array}{cc} 1 & ap_{x}\leq x\leq \left(a-1\right)p_{x},bp_{y}\leq y\leq \left(a-1\right)p_{y}\\ 0 & \text{else} \end{array}\right.$ represents a rectangular function that is equal to 1 in a *p*_*x*_ × *p*_*y*_ rec-tangle about the center $\left(\left(a-1/2\right)\cdot p_{x},y-\left(b-1/2\right)\cdot p_{y}\right)$ and equal to 0 at other locations. ${\text{comb}}_{p_{x},p_{y}}\left(x,y\right)=\sum_{l=-\infty }^{+\infty }\sum_{p=-\infty }^{+\infty }\delta \left(x-\left(l-1/2\right)\cdot p_{x}\right)\cdot \delta \left(y-\left(p-1/2\right)\cdot p_{y}\right)$ represents a comb function. $\delta \left(x-\left(l-1/2\right)\cdot p_{x}\right)=\left\{ \begin{array}{cc} 1 & x=\left(l-1/2\right)\cdot p_{x}\\ 0 & \text{else} \end{array}\right.,\delta \left(y-\left(p-1/2\right)\cdot p_{y}\right)=\left\{ \begin{array}{cc} 1 & y=\left(p-1/2\right)\cdot p_{y}\\ 0 & \text{else} \end{array}\right.$ are the Dirac delta functions. The convolution (denoted by ⊗ ) of the expression in the parentheses of Eq. (3) with the 2D comb function can be understood as a 2D infinite periodic signal generated by the comb function. Therefore, *J*_(*x*,*y*)_ is a periodic function. According to the Fourier series, the 2D function *J*_(*x*,*y*)_, which has a period of *Ap*_*x*_ in the *x*-direction and a period of *Bp*_*y*_ in the *y*-direction, can be expanded as

(4) $$J_{\left(x,y\right)}=\sum_{m=-\infty }^{+\infty }\sum_{n=-\infty }^{+\infty }J_{\left(m,n\right)}\exp\left(\frac{i2\pi mx}{Ap_{x}}\right)\exp\left(\frac{i2\pi ny}{Bp_{y}}\right),$$

where

(5) $$J_{\left(m,n\right)}=\frac{1}{Ap_{x}}\frac{1}{Bp_{y}}\int_{-\frac{Ap_{x}}{2}}^{\frac{Ap_{x}}{2}}\int_{-\frac{Bp_{y}}{2}}^{\frac{Bp_{y}}{2}}J_{\left(x,y\right)}\exp\left(-\frac{i2\pi mx}{Ap_{x}}\right)\exp\left(-\frac{i2\pi ny}{Bp_{y}}\right)dxdy\;\;\;\;\;\;\;\;m,n=0,\pm 1,\pm 2,\ldots$$

Here, *J*_(*m*,*n*)_ is given by the projection of *J*_(*x*,*y*)_ onto the (*m*, *n*) order (the (*m*, *n*) term in the Fourier series of the function *J*_(*x*,*y*)_), which also represents the (*m*, *n*)th transmission coefficient of transmitted light.

On substituting Eq. (4) in Eq. (5), we can obtain

(6) $$\begin{array}{c} J_{\left(m,n\right)}=\frac{1}{Ap_{x}}\frac{1}{Bp_{y}}\int_{-\frac{Ap_{x}}{2}}^{\frac{Ap_{x}}{2}}\int_{-\frac{Bp_{y}}{2}}^{\frac{Bp_{y}}{2}}\left[\left(\sum_{a=1}^{A}\sum_{b=1}^{B}J_{\left(a,b\right)}{\text{rect}}_{p_{x},p_{y}}\left(x-\left(a-1/2\right)\cdot p_{x},y-\left(b-1/2\right)\cdot p_{y}\right)\right)⊗{\text{comb}}_{p_{x},p_{y}}\left(x,y\right)\right]\exp\left(-\frac{i2\pi mx}{Ap_{x}}\right)\exp\left(-\frac{i2\pi ny}{Bp_{y}}\right)dxdy\\ =\frac{1}{A}\frac{1}{B}\sum_{a=1}^{A}\sum_{b=1}^{B}J_{\left(a,b\right)}\sin c\frac{m}{A}\sin c\frac{n}{B}\exp\left(-\frac{i2\pi m\left(a-1/2\right)}{A}\right)\exp\left(-\frac{i2\pi n\left(b-1/2\right)}{B}\right), \end{array}$$

where sinc(x)=sin(πx)/(πx) . The detailed derivation is provided in Section 1, Supplementary material.

## 3 Results and discussion

### 3.1 Conditions for establishing the generalized law of refraction under the incidence of different polarized light

Using Eq. (6), the (*m*, *n*)th transmission coefficient of the transmitted light can be calculated. Subsequently, the anomalous refraction efficiency of the transmitted wave to the (*m*, *n*)th order can be obtained. It should be emphasized that, because the metasurfaces are composed of nanopillars whose Jones matrix is a linear birefringent, unitary matrix, the responses of the metasurfaces to different polarized light vary; consequently, the metasurfaces shape different polarized light in different manners. Thus, *J*_(*m*,*n*)_ is a function related to the polarization states of the incident light; for a certain polarization state of the incident light |q〉 , the anomalous refraction efficiency of the transmitted wave to the (*m*, *n*)th order can be expressed as

(7) $$I^{\left(m,n\right)}=〈q|J_{\left(m,n\right)}^{†}J_{\left(m,n\right)}|q〉\text{ ,}$$

where $J_{\left(m,n\right)}^{†}$ is the conjugate matrix of *J*_(*m*,*n*)_. Furthermore, according to Eqs. (2), (6), and (7), we can deduce the highest achievable anomalous refraction efficiency for the incident light with a certain polarization state transmitted through the metasurfaces to the (*m*, *n*)th transmission order and also the conditions needed when the highest efficiency is reached (that is, the conditions for establishing the generalized law of refraction). These conditions are expected to serve as specific rules for the subsequent design of efficient beam deflection metasurfaces.

Here, we analyze the cases under the incidence of arbitrary linear polarized light, left-handed circularly polarized light, and right-handed circularly polarized light separately.

(a). Arbitrary linear polarized light:

On substituting Eq. (2) and $|q〉=\left[\begin{array}{l} \cos\;\alpha \\ \sin\;\alpha \end{array}\right]$ (*α* is the polarization angle of the linearly-polarized light) in Eqs. (6) and (7), we note that, when the left-hand side of Eq. (8) reaches the maximum (i.e., when Eq. (8) is satisfied), *I* ^(*m*,*n*)^ reaches the maximum value, and the expression of the maximum value is Eq. (9). Herein, *a*, *b*, *c*, and *d* in the equations of this study are all integers, and 1≤a≤A,1≤b≤B,1≤c≤A,1≤d≤B .

(8) $$\begin{array}{l} \cos\left(\theta_{\left(a,b\right)}-\theta_{\left(c,d\right)}\right)\cos\left(\theta_{\left(a,b\right)}+\alpha \right)\cos\left(\theta_{\left(c,d\right)}+\alpha \right)\cos\left(\phi_{x\left(a,b\right)}-\phi_{x\left(c,d\right)}+\frac{2\pi }{A}\left(c-a\right)m+\frac{2\pi }{B}\left(d-b\right)n\right)\\ -\sin\left(\theta_{\left(a,b\right)}-\theta_{\left(c,d\right)}\right)\cos\left(\theta_{\left(a,b\right)}+\alpha \right)\sin\left(\theta_{\left(c,d\right)}+\alpha \right)\cos\left(\phi_{x\left(a,b\right)}-\phi_{y\left(c,d\right)}+\frac{2\pi }{A}\left(c-a\right)m+\frac{2\pi }{B}\left(d-b\right)n\right)\\ +\sin\left(\theta_{\left(a,b\right)}-\theta_{\left(c,d\right)}\right)\sin\left(\theta_{\left(a,b\right)}+\alpha \right)\cos\left(\theta_{\left(c,d\right)}+\alpha \right)\cos\left(\phi_{y\left(a,b\right)}-\phi_{x\left(c,d\right)}+\frac{2\pi }{A}\left(c-a\right)m+\frac{2\pi }{B}\left(d-b\right)n\right)\\ +\cos\left(\theta_{\left(a,b\right)}-\theta_{\left(c,d\right)}\right)\sin\left(\theta_{\left(a,b\right)}+\alpha \right)\sin\left(\theta_{\left(c,d\right)}+\alpha \right)\cos\left(\phi_{y\left(a,b\right)}-\phi_{y\left(c,d\right)}+\frac{2\pi }{A}\left(c-a\right)m+\frac{2\pi }{B}\left(d-b\right)n\right)\\ =1 \end{array}$$

(9) $$I_{\max}^{\left(m,n\right)}=\sin c^{2}\frac{m}{A}\sin c^{2}\frac{n}{B}$$

**Figure 3:**
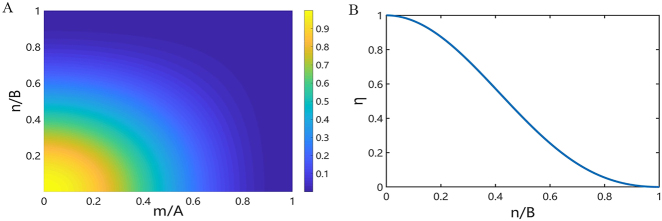
(A) The relationship between the highest achievable anomalous refraction efficiency and *m*/*A*, *n*/*B* when the incident light is diffracted to the (*m*, *n*)th order. (B) The relationship between the highest achievable anomalous refraction efficiency and *n*/*B* when the incident light is diffracted to the (0, *n*)th order and when the metasurface has only one phase gradient along a single direction.

According to Eq. (9), we can obtain the relationship between the highest achievable anomalous refraction efficiency and *m*/*A*, *n*/*B* when the incident light is diffracted through the metasurfaces to the (*m*, *n*)th order (Figure 3A). It can be seen that the higher the diffraction order and the smaller the number of nanoparticles in a single supercell of the metasurface, the lower the diffraction efficiency, and the diffraction efficiency cannot reach 100%. When ${\left(\frac{m}{A}\right)}^{2}+{\left(\frac{n}{B}\right)}^{2}\leq 0.2573^{2}$ , the maximum achievable efficiency of the incident light diffracted through the metasurface to the (*m*, *n*)th order can be greater than 80%. Therefore, when designing an efficient deflection metasurface, the ratio of the diffraction orders to the number of nanoparticles in a single supercell of the metasurface needs to be carefully considered.

According to Eq. (8), a special case can be observed. When *ϕ*_*x*(*a*,*b*)_, *ϕ*_*x*(*c*,*d*)_, *ϕ*_*y*(*a*,*b*)_, and *ϕ*_*y*(*c*,*d*)_ satisfy Eq. (10), irrespective of the values of *θ*_(*a*,*b*)_ and *θ*_(*c*,*d*)_, Eq. (8) always holds.

(10) $$\begin{array}{c} \cos\left(\phi_{x\left(a,b\right)}-\phi_{x\left(c,d\right)}+\frac{2\pi }{A}\left(c-a\right)m+\frac{2\pi }{B}\left(d-b\right)n\right)=1\\ \cos\left(\phi_{y\left(a,b\right)}-\phi_{y\left(c,d\right)}+\frac{2\pi }{A}\left(c-a\right)m+\frac{2\pi }{B}\left(d-b\right)n\right)=1\\ \cos\left(\phi_{y\left(a,b\right)}-\phi_{x\left(c,d\right)}+\frac{2\pi }{A}\left(c-a\right)m+\frac{2\pi }{B}\left(d-b\right)n\right)=1\\ \cos\left(\phi_{x\left(a,b\right)}-\phi_{y\left(c,d\right)}+\frac{2\pi }{A}\left(c-a\right)m+\frac{2\pi }{B}\left(d-b\right)n\right)=1 \end{array}$$

In addition, according to Eq. (8) and when the nanopillars in the metasurfaces have no rotation angles (i.e., the phase change (*ϕ*_*x*_, *ϕ*_*y*_) introduced by each nanopillar in the metasurfaces to different polarized light only depends on the propagation phase), we obtain the conditions that the metasurfaces need to meet in order to realize the maximum value of *I* ^(*m*,*n*)^. In this case, Eq. (2) can be simplified as

(11) $$J_{\left(a,b\right)}=[\begin{array}{l} e^{i\phi_{x\left(a,b\right)}}\\ 0 \end{array}\begin{array}{r} 0\\ e^{i\phi_{y\left(a,b\right)}} \end{array}].$$

On substituting Eq. (11) and $|q〉=\left[\begin{array}{l} \cos\alpha \\ \sin\alpha \end{array}\right]$ in Eqs. (6) and (7) (or when *θ*_(*a*,*b*)_ = 0 and *θ*_(*c*,*d*)_ = 0 in Eq. (8)), we note that when Eq. (12) is satisfied, *I* ^(*m*,*n*)^ reaches the maximum value, and the expression of this maximum value is still Eq. (9).

(12) $$\begin{array}{l} \phi_{x\left(1,1\right)}-\phi_{x\left(a,b\right)}+\frac{2\pi }{A}\left(a-1\right)m+\frac{2\pi }{B}\left(b-1\right)n=2k\pi \;\;\;\;\;k=0,\pm 1,\pm 2\ldots ,\\ \phi_{y\left(1,1\right)}-\phi_{y\left(a,b\right)}+\frac{2\pi }{A}\left(a-1\right)m+\frac{2\pi }{B}\left(b-1\right)n=2k\pi \;\;\;\;\;k=0,\pm 1,\pm 2\ldots , \end{array}$$

In particular, when the metasurface has only one phase gradient along a single direction, that is, *A*/*B* = 1, Eq. (12) can be simplified. For example, when *A* = 1, Eq. (12) can be simplified as

(13) $$\begin{array}{c} \phi_{x\left(1,1\right)}-\phi_{x\left(1,b\right)}+\frac{2\pi }{B}\left(b-1\right)n=2k\pi \;\;\;\;\;k=0,\pm 1,\pm 2\ldots ,\\ \phi_{y\left(1,1\right)}-\phi_{y\left(1,b\right)}+\frac{2\pi }{B}\left(b-1\right)n=2k\pi \;\;\;\;\;k=0,\pm 1,\pm 2\ldots . \end{array}$$

This is the most commonly designed structure for beam deflection transmissive metasurfaces [28], [29], [30], [31]. This type of metasurface typically fails to achieve high-order diffraction in the *x*-direction, and only the (0, 0)th diffraction is realized in the *x*-direction (i.e., *m* is equal to zero). This is because, in this case, generally, the supercell period of the metasurfaces in the *x*-direction is smaller than the wavelength of the incident wave. Thus, Eq. (9) can be simplified to

(14) $$I_{\max}^{\left(m,n\right)}=\sin c^{2}\frac{n}{B}.$$

According to Eq. (14), we can obtain the relationship between the highest achievable efficiency and *n*/*B* when the incident light is diffracted through the metasurfaces to the (0, *n*)th order (Figure 3B). It can also be seen that the higher the diffraction order and the smaller the number of nanoparticles in a single supercell of the metasurface, the lower the diffraction efficiency, and the diffraction efficiency cannot reach 100%. When *n*/*B* ≤ 0.2573, the maximum achievable efficiency of the incident light diffracted through the metasurface to the (0, *n*)th order can be greater than 80%. Therefore, when the ratio of the diffraction order to the number of nanoparticles in a single supercell of the metasurface is no more than 0.2573, it is possible to design a metasurface with an anomalous refraction efficiency higher than 80%.

There are two particular cases:

1. When *α* = 0°, that is when the incident light is 0° linear polarized incident light, Eqs. (12) and (13) can be simplified to

(15) $$\phi_{x\left(1,1\right)}-\phi_{x\left(a,b\right)}+\frac{2\pi }{A}\left(a-1\right)m+\frac{2\pi }{B}\left(b-1\right)n=2k\pi \;k=0,\pm 1,\pm 2\ldots$$

(16) $$\phi_{x\left(1,1\right)}-\phi_{x\left(1,b\right)}+\frac{2\pi }{B}\left(b-1\right)n=2k\pi \;k=0,\pm 1,\pm 2\ldots$$

In addition, we emphasize that, in this case, the generalized law of refraction is established under the condition that the phase change, *ϕ*_*x*(*a*,*b*)_, introduced by the nanopillars over one period of the metasurface to the 0° linear polarized incident light satisfies Eq. (15). This is different to previous reports [8], [9], [10], [11], [12], [13], [14], [15], [16], [17], [18], where the nanopillars in one period of the metasurface need to introduce a linear distribution phase gradient in the 2*π* range.

(2) When *α* = 90°, that is when the incident light is 90° linear polarized incident light, Eqs. (12) and (13) can be simplified to

(17) $$\phi_{y\left(1,1\right)}-\phi_{y\left(a,b\right)}+\frac{2\pi }{A}\left(a-1\right)m+\frac{2\pi }{B}\left(b-1\right)n=2k\pi \;k=0,\pm 1,\pm 2\ldots$$

(18) $$\phi_{y\left(1,1\right)}-\phi_{y\left(1,b\right)}+\frac{2\pi }{B}\left(b-1\right)n=2k\pi \;k=0,\pm 1,\pm 2\ldots$$

(b). Left-handed circularly polarized light:

On substituting Eq. (2) and $|q〉=\frac{\sqrt{2}}{2}\left[\begin{array}{l} 1\\ i \end{array}\right]$ in Eqs. (6) and (7), we note that, when the left-hand side of Eq. (19) reaches the maximum (i.e., when Eq. (19) is satisfied), *I* ^(*m*,*n*)^ reaches the maximum value, and the expression of the maximum value is the same as Eq. (9).

According to Eq. (19), a special case can be identified, that is, when *ϕ*_*x*(*a*,*b*)_, *ϕ*_*x*(*c*,*d*)_, *ϕ*_*y*(*a*,*b*)_, and *ϕ*_*y*(*c*,*d*)_ satisfy Eq. (10), irrespective of the values of *θ*_(*a*,*b*)_ and *θ*_(*c*,*d*)_, Eq. (19) always holds.

In addition, when the nanopillars in the metasurfaces have no rotation angles, on substituting Eq. (11) and $|q〉=\frac{\sqrt{2}}{2}\left[\begin{array}{l} 1\\ i \end{array}\right]$ in Eqs. (6) and (7) (or when *θ*_(*a*,*b*)_ = 0 and *θ*_(*c*,*d*)_ = 0 in Eq. (19)), we obtain the same case as that under the incidence of any linear polarized light. In other words, when Eq. (12) is satisfied, *I* ^(*m*,*n*)^ reaches the maximum value, and the expression of the maximum value is the same as Eq. (9).

(19) $$\begin{array}{l} \cos^{2}\left(\theta_{\left(a,b\right)}-\theta_{\left(c,d\right)}\right)\cos\left(\phi_{x\left(a,b\right)}-\phi_{x\left(c,d\right)}+\frac{2\pi }{A}\left(c-a\right)m+\frac{2\pi }{B}\left(d-b\right)n\right)+\sin^{2}\left(\theta_{\left(a,b\right)}-\theta_{\left(c,d\right)}\right)\cos\left(\phi_{x\left(a,b\right)}-\phi_{y\left(c,d\right)}+\frac{2\pi }{A}\left(c-a\right)m+\frac{2\pi }{B}\left(d-b\right)n\right)\\ +\sin^{2}\left(\theta_{\left(a,b\right)}-\theta_{\left(c,d\right)}\right)\cos\left(\phi_{y\left(a,b\right)}-\phi_{x\left(c,d\right)}+\frac{2\pi }{A}\left(c-a\right)m+\frac{2\pi }{B}\left(d-b\right)n\right)+\cos^{2}\left(\theta_{\left(a,b\right)}-\theta_{\left(c,d\right)}\right)\cos\left(\phi_{y\left(a,b\right)}-\phi_{y\left(c,d\right)}+\frac{2\pi }{A}\left(c-a\right)m+\frac{2\pi }{B}\left(d-b\right)n\right)\\ +\cos\left(\theta_{\left(a,b\right)}-\theta_{\left(c,d\right)}\right)\sin\left(\theta_{\left(a,b\right)}-\theta_{\left(c,d\right)}\right)\sin\left(\phi_{x\left(a,b\right)}-\phi_{x\left(c,d\right)}+\frac{2\pi }{A}\left(c-a\right)m+\frac{2\pi }{B}\left(d-b\right)n\right)\\ +\cos\left(\theta_{\left(a,b\right)}-\theta_{\left(c,d\right)}\right)\sin\left(\theta_{\left(a,b\right)}-\theta_{\left(c,d\right)}\right)\sin\left(\phi_{y\left(a,b\right)}-\phi_{y\left(c,d\right)}+\frac{2\pi }{A}\left(c-a\right)m+\frac{2\pi }{B}\left(d-b\right)n\right)\\ -\cos\left(\theta_{\left(a,b\right)}-\theta_{\left(c,d\right)}\right)\sin\left(\theta_{\left(a,b\right)}-\theta_{\left(c,d\right)}\right)\sin\left(\phi_{x\left(a,b\right)}-\phi_{y\left(c,d\right)}+\frac{2\pi }{A}\left(c-a\right)m+\frac{2\pi }{B}\left(d-b\right)n\right)\\ -\cos\left(\theta_{\left(a,b\right)}-\theta_{\left(c,d\right)}\right)\sin\left(\theta_{\left(a,b\right)}-\theta_{\left(c,d\right)}\right)\sin\left(\phi_{y\left(a,b\right)}-\phi_{x\left(c,d\right)}+\frac{2\pi }{A}\left(c-a\right)m+\frac{2\pi }{B}\left(d-b\right)n\right)\\ =2 \end{array}$$

When the metasurface has only one phase gradient along a single direction, such as when *A* = 1, the same as the case under the incidence of any linear polarized light, when Eq. (13) is satisfied, *I* ^(*m*,*n*)^ reaches the maximum value, and the expression of the maximum value is simplified to Eq. (14).

According to Eq. (19) and when only changing the rotation angles of the nanopillars (which implies that the phase variation introduced by each nanopillar depends on the geometric phase alone), the conditions that the metasurfaces need to meet for realizing the maximum value of *I* ^(*m*,*n*)^ can be analyzed. In this case, Eq. (2) can be simplified as

(20) $$J_{\left(a,b\right)}=R\left(-\theta_{\left(a,b\right)}\right)[\begin{array}{l} e^{i\phi_{x}}\\ 0 \end{array}\begin{array}{r} 0\\ e^{i\phi_{y}} \end{array}]R\left(\theta_{\left(a,b\right)}\right).$$

On substituting Eq. (20) and $|q〉=\frac{\sqrt{2}}{2}\left[\begin{array}{l} 1\\ i \end{array}\right]$ in Eqs. (6) and (7) (or when *ϕ*_*x*(*a*,*b*)_ = *ϕ*_*x*(*c*,*d*)_ and *ϕ*_*y*(*a*,*b*)_ = *ϕ*_*y*(*c*,*d*)_ in Eq. (19)), we note that *I* ^(*m*,*n*)^ reaches the maximum value when $\phi_{x}-\phi_{y}=\pm \pi$ in Eqs. (20) and (21) is satisfied; the expression of the maximum value is still Eq. (9). The detailed derivation is presented in Section 2, Supplementary material.

(21) $$\cos[2\left(\theta_{\left(a,b\right)}-\theta_{\left(c,d\right)}\right)-\frac{2\pi }{A}\left(c-a\right)m-\frac{2\pi }{B}\left(d-b\right)n]=1$$

(c). Right-handed circularly polarized light:

On substituting Eq. (2) and $|q〉=\frac{\sqrt{2}}{2}\left[\begin{array}{l} 1\\ -i \end{array}\right]$ in Eqs. (6) and (7), we note that, when the left-hand side of Eq. (22) reaches the maximum (i.e., when Eq. (22) is satisfied), *I* ^(*m*,*n*)^ reaches the maximum value, and the expression of the maximum value is also Eq. (9).

(22) $$\begin{array}{l} \cos^{2}\left(\theta_{\left(a,b\right)}-\theta_{\left(c,d\right)}\right)\cos\left(\phi_{x\left(a,b\right)}-\phi_{x\left(c,d\right)}+\frac{2\pi }{A}\left(c-a\right)m+\frac{2\pi }{B}\left(d-b\right)n\right)+\sin^{2}\left(\theta_{\left(a,b\right)}-\theta_{\left(c,d\right)}\right)\cos\left(\phi_{x\left(a,b\right)}-\phi_{y\left(c,d\right)}+\frac{2\pi }{A}\left(c-a\right)m+\frac{2\pi }{B}\left(d-b\right)n\right)\\ +\sin^{2}\left(\theta_{\left(a,b\right)}-\theta_{\left(c,d\right)}\right)\cos\left(\phi_{y\left(a,b\right)}-\phi_{x\left(c,d\right)}+\frac{2\pi }{A}\left(c-a\right)m+\frac{2\pi }{B}\left(d-b\right)n\right)+\cos^{2}\left(\theta_{\left(a,b\right)}-\theta_{\left(c,d\right)}\right)\cos\left(\phi_{y\left(a,b\right)}-\phi_{y\left(c,d\right)}+\frac{2\pi }{A}\left(c-a\right)m+\frac{2\pi }{B}\left(d-b\right)n\right)\\ -\cos\left(\theta_{\left(a,b\right)}-\theta_{\left(c,d\right)}\right)\sin\left(\theta_{\left(a,b\right)}-\theta_{\left(c,d\right)}\right)\sin\left(\phi_{x\left(a,b\right)}-\phi_{x\left(c,d\right)}+\frac{2\pi }{A}\left(c-a\right)m+\frac{2\pi }{B}\left(d-b\right)n\right)\\ -\cos\left(\theta_{\left(a,b\right)}-\theta_{\left(c,d\right)}\right)\sin\left(\theta_{\left(a,b\right)}-\theta_{\left(c,d\right)}\right)\sin\left(\phi_{y\left(a,b\right)}-\phi_{y\left(c,d\right)}+\frac{2\pi }{A}\left(c-a\right)m+\frac{2\pi }{B}\left(d-b\right)n\right)\\ +\cos\left(\theta_{\left(a,b\right)}-\theta_{\left(c,d\right)}\right)\sin\left(\theta_{\left(a,b\right)}-\theta_{\left(c,d\right)}\right)\sin\left(\phi_{x\left(a,b\right)}-\phi_{y\left(c,d\right)}+\frac{2\pi }{A}\left(c-a\right)m+\frac{2\pi }{B}\left(d-b\right)n\right)\\ +\cos\left(\theta_{\left(a,b\right)}-\theta_{\left(c,d\right)}\right)\sin\left(\theta_{\left(a,b\right)}-\theta_{\left(c,d\right)}\right)\sin\left(\phi_{y\left(a,b\right)}-\phi_{x\left(c,d\right)}+\frac{2\pi }{A}\left(c-a\right)m+\frac{2\pi }{B}\left(d-b\right)n\right)\\ =2 \end{array}$$

According to Eq. (22), a special case can be identified, that is, when *ϕ*_*x*(*a*,*b*)_, *ϕ*_*x*(*c*,*d*)_, *ϕ*_*y*(*a*,*b*)_, and *ϕ*_*y*(*c*,*d*)_ satisfy Eq. (10), irrespective of the values of *θ*_(*a*,*b*)_ and *θ*_(*c*,*d*)_, Eq. (22) always holds.

In addition, when the nanopillars in the metasurface have no rotation angles, on substituting Eq. (11) and $|q〉=\frac{\sqrt{2}}{2}\left[\begin{array}{l} 1\\ -i \end{array}\right]$ in Eqs. (6) and (7) (or when *θ*_(*a*,*b*)_ = 0 and *θ*_(*c*,*d*)_ = 0 in Eq. (22)), we obtain the same case as that under the incidence of any linear polarized light. In other words, when Eq. (12) is satisfied, *I* ^(*m*,*n*)^ reaches the maximum value, and the expression of the maximum value is Eq. (9). When the metasurface has only one phase gradient along a single direction, such as when *A* = 1, the same as the case under the incidence of any linear polarized light, when Eq. (13) is satisfied, *I* ^(*m*,*n*)^ reaches the maximum value, and the expression of the maximum value is simplified to Eq. (14).

When only changing the rotation angles of the nanopillars, on substituting Eq. (22) and $|q〉=\frac{\sqrt{2}}{2}\left[\begin{array}{l} 1\\ -i \end{array}\right]$ in Eqs. (6) and (7) (or when *ϕ*_*x*(*a*,*b*)_ = *ϕ*_*x*(*c*,*d*)_ and *ϕ*_*y*(*a*,*b*)_ = *ϕ*_*y*(*c*,*d*)_ in Eq. (22)), we note that *I* ^(*m*,*n*)^ reaches the maximum value when $\phi_{x}-\phi_{y}=\pm \pi$ in Eqs. (20) and (23) Eq. (20), and Eq. (23) is satisfied; the expression of the maximum value is the same as Eq. (9). The detailed derivation is presented in Section 2, Supplementary material.

(23) $$\cos[2\left(\theta_{\left(a,b\right)}-\theta_{\left(c,d\right)}\right)+\frac{2\pi }{A}\left(c-a\right)m+\frac{2\pi }{B}\left(d-b\right)n]=1$$

### 3.2 Verification

First, we prove the accuracy of the derivation process (i.e., the accuracy of the derived Eqs. (8), (9), (12), (15), (17), (19), (21), (22) and (23) when the model Eqs. (2), (6), (7) are established). We substitute Eq. (2) and $|q〉=\left[\begin{array}{l} 1\\ 0 \end{array}\right]$ , $|q〉=\left[\begin{array}{c} \frac{\sqrt{3}}{2}\\ \frac{1}{2} \end{array}\right]$ , $|q〉=\left[\begin{array}{l} \cos\frac{3\pi }{5}\\ \sin\frac{3\pi }{5} \end{array}\right]$ , or $|q〉=\frac{\sqrt{2}}{2}\left[\begin{array}{l} 1\\ -1 \end{array}\right]$ respectively in Eqs. (6) and (7) and calculate the phase changes that need to be introduced by the nanopillars to different polarized incident light and the rotation angles of the nanopillars when *I* ^(*m*,*n*)^ reaches the maximum value, by using the interior point method. The case considered involves *A* = 1, *B* = 5, and (*m*, *n*) = (0, 1). The calculation results are listed in columns 2–5 of Table S1, Supplementary material, and the values satisfy Eq. (8) (when *α* = 0°, 30°, 45°, 108° respectively). We also substitute Eq. (2) and $|q〉=\frac{\sqrt{2}}{2}\left[\begin{array}{l} 1\\ i \end{array}\right]$ or $|q〉=\frac{\sqrt{2}}{2}\left[\begin{array}{l} 1\\ -i \end{array}\right]$ respectively in Eqs. (6) and (7) and calculate the phase changes that need to be introduced by the nanopillars to different polarized incident light and the rotation angles of the nanopillars when *I* ^(*m*,*n*)^ reaches the maximum value, by using the interior point method. The case considered involves *A* = 1, *B* = 5, and (*m*, *n*) = (0, 1). The calculation results are listed in columns 6–7 of Table S1, Supplementary material, and the values satisfy Eqs. (19) and (22) respectively. It should be noted that Table S1 only provides one type of results. When the phase variations introduced by the nanopillar at the position of (1, 1) vary, the phase variations introduced by the nanoparticles at other positions will also change; however, the difference between the phase variations introduced by the nanoparticles at different positions will still satisfy Eqs. (8), (19) and (22) respectively. We also calculate the highest achievable efficiency of anomalous refraction to the (1, 1)th order under the incidence of different polarized light for the cases of *A* = 2 and *B* = 2, *A* = 3 and *B* = 3, *A* = 4 and *B* = 4, *A* = 5 and *B* = 5, or *A* = 6 and *B* = 6, by using the interior point method (see Table S2 and Figure S1, Supplementary material). It is evident that the maximum efficiency values are basically equal to the theoretical ones calculated using Eq. (9). The reason for the slightly smaller values than the theoretical ones of the cases of *A* = 5 and *B* = 5 and *A* = 6 and *B* = 6 is that the initial values cannot be accurately selected when calculating the maximum values by the interior point method.

To verify the accuracy of Eqs. (12), (15), and (17), we substitute Eq. (11) and $|q〉=\left[\begin{array}{l} 1\\ 0 \end{array}\right]$ , $|q〉=\left[\begin{array}{l} 0\\ 1 \end{array}\right]$ , $|q〉=\frac{\sqrt{2}}{2}\left[\begin{array}{l} 1\\ 1 \end{array}\right]$ , or $|q〉=\frac{\sqrt{2}}{2}\left[\begin{array}{l} 1\\ -1 \end{array}\right]$ respectively in Eqs. (6) and (7), and then calculate the phase changes that need to be introduced by the nanopillars to different polarized incident light when *I* ^(*m*,*n*)^ reaches the maximum value, by using the interior point method. The case considered is *A* = 5, *B* = 5, and (*m*, *n*) = (1, 1). The calculation results are listed in Table S3, Supplementary material, and the values in columns 2–5 of Table S3 satisfy Eqs. (12), (15), and (17) respectively.

To verify the accuracy of Eqs. (21) and (23), we substitute Eq. (20) and $|q〉=\frac{\sqrt{2}}{2}\left[\begin{array}{l} 1\\ i \end{array}\right]$ or $|q〉=\frac{\sqrt{2}}{2}\left[\begin{array}{l} 1\\ -i \end{array}\right]$ respectively in Eqs. (6) and (7) and then calculate the phase changes that need to be introduced by the nanopillars to different circularly polarized incident light and the rotation angles of the nanopillars when *I* ^(*m*,*n*)^ reaches the maximum value, by using the interior point method. The case considered is *A* = 1, *B* = 5, and (*m*, *n*) = (0, 1). The calculation results are presented in Table S4, Supplementary material, and the values in columns 2–3 of Table S4 satisfy Eqs. (21) and (23) respectively.

To verify the accuracy of the established model (Eqs. (2), (6), and (7)) in design, we numerically simulate the anomalous refraction efficiency for different polarized incident light transmitted to the corresponding (*m*, *n*)th order after passing through the metasurfaces designed according to the above-mentioned equations, through FDTD Solutions. The basic dimensions of the metasurface unit cell are as follows: the periods in the *x*- and *y*-directions (i.e., *p*_*x*_ and *p*_*y*_, respectively) are both 500 nm, the height of the nanopillars is 500 nm, the thickness of the substrate is 200 nm, the material of the nanopillars is set as the Si (Palik) model in FDTD, and the substrate material is set as SiO_2_ with a refractive index of 1.46. The specific verifications are detailed below.

First, we verify the case when the nanopillars have no rotation angles. As can be seen from Eqs. (12), (15), and (17), Eq. (12) contains Eqs. (15) and (17). Therefore, if Eq. (12) is satisfied, the anomalous refraction efficiency will reach the maximum value under the incidence of any polarized light. The case considered is *A* = 5, *B* = 5, and (*m*, *n*) = (1, 1), and the incident wavelength is 1300 nm. The phase variations that need to be introduced to different polarized incident light are presented in columns 4 of Table S3 calculated according to Eq. (12), by using the interior point method. We select the appropriate nanopillars according to the phase values. The cross-sections of the nanopillars selected are all square because, according to the data in columns 4 of Table S3, *ϕ*_*x*(*a*,*b*)_ = *ϕ*_*y*(*a*,*b*)_. The specific dimensions of the selected nanopillars, the transmission efficiency through the nanopillars under the incidence of 0° linear polarized light, and the actual phase variations introduced by the selected nanopillars are presented in Table S5, Supplementary material. Through the simulations in FDTD Solutions, we obtain the anomalous refraction efficiency of different polarized incident light transmitted to the (1, 1)th order through the metasurfaces composed by the selected nanopillars. The results are shown in Table S6 and Figure S2, Supplementary material. It is evident that the efficiency is basically consistent with the theoretically achievable maximum efficiency, obtained according to Eq. (9). The lower efficiency in certain cases is likely because the transmission efficiency through the nanopillars under the incidence of 0° linear polarized light is low (it also means that these cases fail to meet the limitations in Section 2, that is, the amplitude of the incident light is also adjusted by the nanopillars) and the scattering between the nanopillars interferes. We also provide the simulated corresponding electric field component field patterns, far-field electric field intensity as a function of the diffraction angle and far field electric field distribution respectively (Figures S3–S5, Supplementary material). The results prove the high anomalous refraction efficiency of different polarized incident light transmitted to the (1, 1)th order through the metasurfaces, and the deflection angle estimated (azimuth angle (*φ*) = 45°, zenith angle (*θ*) = 45°) in simulation (Figure S4) is also consistent with the theoretical value of which is derived from the well-known generalized Snell’s law (*φ = θ = *√2 * arcsin(*λ*/B/*p*_*y*_) * 180/*π* = 44.3°). We also evaluate the case when *A* = 1, *B* = 5, (*m*, *n*) = (0, 1), the incident light is 1300 nm 0° linear polarized light, and nanopillars have no rotation angles. Here, we compare two design methods: designing the metasurface based on the phase data calculated according to Eq. (15) by using the interior point method and designing the metasurface based on the phase values linearly selected from 0 to 2*π*. The sizes of the selected nanopillars, the transmission efficiency of the 0° linear polarized incident light through the nanopillars, the calculated phase variations that need to be introduced by the nanopillars, and the actual phase variations introduced by the selected nanopillars of the two methods are presented in Table S7 and S8, Supplementary material. Through the simulations in FDTD Solutions, the anomalous refraction efficiency values for 0° linear polarized incident light passing through the two metasurfaces composed by the selected nanopillars to the (0, 1)th order are obtained. The results are shown in Table S9 and Figure S6, Supplementary material. As is evident, the efficiency obtained by the first design method is basically consistent with the one obtained according to Eq. (14) and higher than that obtained using the second design method. This also proves that in this case, the condition for the establishment of the generalized law of refraction is to satisfy Eq. (15), rather than introducing a linear distribution phase gradient within the range of 2*π* using the nanopillars in one period of the metasurface. We also provide the simulated Ex component of the transmitted light in the *yz* plane and far-field electric field intensity as a function of the diffraction angle (Figures S7 and S8, Supplementary material) to prove the high deflection efficiency when using the first design method. The deflection angle estimated (zenith angle (*θ*) = 30.3°) in simulation (Figure S8) is also consistent with the theoretical value of which is derived from the generalized Snell’s law (*θ = *arcsin(*λ*/*B*/*p*_*y*_) * 180/*π* = 31.3°).

We also verify the case where the nanopillars have rotation angles. The case considered is *A* = 1, *B* = 5, (*m*, *n*) = (0, 1), and the incident light is 1300 nm 0° linear polarized light. We select suitable nanopillars from the phase changes calculated according to Eq. (8) by using the interior point method and assign them the calculated rotation angles. The specific sizes of the selected nanopillars, the transmission efficiency through the nanopillars, the calculated phase variations that need to be introduced by the nanopillars, and the actual phase variations introduced by the selected nanopillars are listed in Table S10, Supplementary material. Through the simulations in FDTD Solutions, we obtain the anomalous refraction efficiency for the 0° linear polarized incident light transmitted to the (0, 1)th order through the metasurface composed by the selected nanopillars (total transmission efficiency *T* = 0.8034, transmission efficiency projected to the (0, 1)th order *T*(0, 1) = 0.76, and efficiency of anomalous refraction to the (0, 1)th order *η* = 0.9461). It is evident that the efficiency is basically the same as the theoretically one (*η* = 0.8751) obtained according to Eq. (14). The slightly higher value is likely due to the interference between nanopillars. We also provide the simulated Ex component of the transmitted light in the *yz* plane, far-field electric field intensity as a function of the diffraction angle and far field electric field distribution respectively (Figures S9–S11, Supplementary material) to prove the high deflection efficiency. The deflection angle estimated (zenith angle (*θ*) = 30.3°) in simulation (Figure S10) is also consistent with the theoretical value of which is derived from the generalized Snell’s law (*θ = *arcsin(*λ*/*B*/*p*_*y*_) * 180/*π* = 31.3°). We also calculate the cases under the incidence of 30°, 45°, 108° linear polarized incident light and left-handed circularly polarized incident light (Section 3, Supplementary material).

Lastly, we also verify the case where the size of the nanopillars of the metasurface remains unchanged and only the rotation angles are varied. The case considered is *A* = 1, *B* = 5, (*m*, *n*) = (0, 1), and the incident light is 1200 nm left-handed circularly polarized light. We select suitable nanopillars based on the phase changes calculated according to Eq. (21) by using the interior point method and assign them the calculated rotation angles. The specific sizes of the selected nanopillars, the transmission efficiency through the nanopillars, the calculated phase variations that need to be introduced by the nanopillars, and the actual phase variations introduced by the selected nanopillars are listed in Table S11, Supplementary material. The calculated rotation angles of the nanopillars are presented in Table S12, Supplementary material. Through the simulations in FDTD Solutions, we obtain the anomalous refraction efficiency for the left-handed circularly polarized incident light transmitted to the (0, 1)th order through the metasurface composed by the selected nanopillars (total transmission efficiency *T* = 0.5932, transmission efficiency projected to the (0, 1)th order *T*(0, 1) = 0.5228, and efficiency of anomalous refraction to the (0, 1)th order *η* = 0.8813). It is evident that the efficiency is basically the same as the theoretically one (*η* = 0.8751) obtained according to Eq. (14). The slightly higher value is likely caused by the interference between nanopillars. We also provide the far-field electric field intensity as a function of the diffraction angle and far field electric field distribution, respectively (Figures S17 and S18, Supplementary material). The results prove the high anomalous refraction efficiency of left-handed circularly polarized incident light transmitted to the (0, 1)th order through the metasurfaces, and the deflection angle estimated (zenith angle (*θ*) = 28.5°) in simulation (Figure S17) is also consistent with the theoretical value of which is derived from the generalized Snell’s law (*θ* = arcsin(*λ*/*B*/*p*_*y*_) * 180/*π* = 28.7°). The case of the right-handed circularly polarized incident light is similar to the case of the left-handed circularly polarized incident light; thus, it is not proven herein.

### 3.3 Conditions for establishing polarization-insensitive generalized law of refraction

It can be seen that, when Eq. (10) is satisfied, the efficiency of the anomalous refraction will reach the maximum value under the incidence of any polarized light. The expression for the achievable highest efficiency is Eq. (9). In addition, when the nanopillars in the metasurfaces have no rotation angles, by combining Eqs. (12), (15), and (17), we note that the efficiency of the anomalous refraction will reach the maximum value under the incidence of any polarized light, provided *ϕ*_*x*(*a*,*b*)_ and *ϕ*_*y*(*a*,*b*)_ satisfy Eq. (12); the expression for the highest achievable efficiency is also Eq. (9).

## 4 Discussion

Based on Section 3.1, metasurfaces composed of rotatable nanopillars can be designed with greater freedom and more adjustable parameters; however, the design principles of the metasurfaces composed of nanopillars without rotation angles are simpler and the design process is easier to understand and control.

This study focuses on the conditions that the parameters (*θ*_(*a*,*b*)_, *θ*_(*c*,*d*)_, *ϕ*_*x*(*a*,*b*)_, *ϕ*_*x*(*c*,*d*)_, *ϕ*_*y*(*a*,*b*)_, and *ϕ*_*y*(*c*,*d*)_) need to meet for realizing the maximum anomalous refractive efficiency under the incidence of different polarized light. It also provides theoretical bases for designing high-efficiency beam deflection metasurfaces. However, this research does not involve the selection and optimization of the parameters for the sizes of the metasurfaces. Nevertheless, we identify a general method for selecting and optimizing the parameters. According to the equations that need to be satisfied when the generalized law of refraction is established under the incidence of different polarized light, as given in Section 3.1, the parameters (*θ*_(*a*,*b*)_, *θ*_(*c*,*d*)_, *ϕ*_*x*(*a*,*b*)_, *ϕ*_*x*(*c*,*d*)_, *ϕ*_*y*(*a*,*b*)_, *ϕ*_*y*(*c*,*d*)_) can be calculated and determined using the interior point method or other algorithms. By using numerical simulation methods (such as FDTD Solutions) to calculate the transmission efficiency of the 0° linear polarized incident light through nanopillars with different sizes (*t*_*x*_), the phase variations introduced by the nanopillars of different sizes to the 0° linear polarized incident light $\phi_{x\left(a,b\right)}^{\text{actual}}$ , the transmission efficiency of the 90° linear polarized incident light through the nanopillars of different sizes *t*_*y*_, and the phase variations introduced by the nanopillars of different sizes to the 90° linear polarized incident light $\phi_{y\left(a,b\right)}^{\text{actual}}$ , we can combine these data to form a database and then calculate the minimum of

(28) $${\left|t_{x\left(a,b\right)}e^{i\phi_{x\left(a,b\right)}^{\text{actual}}}-e^{i\phi_{x\left(a,b\right)}}\right|}^{2}+{\left|t_{y\left(a,b\right)}e^{i\phi_{y\left(a,b\right)}^{\text{actual}}}-e^{i\phi_{y\left(a,b\right)}}\right|}^{2},$$

through optimized algorithms. We may then select the appropriate sizes of the nanopillars from this database.

Here, we also note the default condition considered in this study, that is, the number of nanopillars within one period of the metasurface needs to satisfy $\frac{m\lambda_{in}}{Ap_{x}}\leq 1,\frac{n\lambda_{in}}{Bp_{y}}\leq 1$ . In addition, according to Eq. (9), when *A* and *B* are larger, the maximum value of the achievable anomalous refraction efficiency is higher. However, increasing *A* and *B* will lead to an increase in the difficulty of selecting the structural parameters for the metasurfaces and an increase in the difficulty of production. Therefore, it is necessary to balance the tradeoff between the two factors in order to determine the most suitable number of nanopillars within one period of the metasurface. In addition, this study has a few limitations that warrant further research. 1. This study assumes that the interaction between the nanoparticles and light is limited to the inside of the particles; however, in practical scenarios, the scattering of a single nanopillar is occasionally severely affected by the scattering of the surrounding particles. Therefore, it is important to further study the anomalous refraction efficiency while considering the influence of the scattering of surrounding particles. 2. Although we mentioned that we currently only focus on metasurfaces composed by the unit cell containing one nanoparticle, our work are still applicable to the metasurfaces which are composed of multipole nanopillars in one unit cell [32], [33], [34], as long as their Jones matrixes act as linear birefringent, unitary matrixes and can be represented as Eq. (1). However, at that time it should be assumed that the interaction between the unit cells and light is limited within the unit cells and is not affected by the scattering of the surrounding unit cells. 3. The condition of normal incidence is considered in this study. However, to roughly study the anomalous refraction performance of the designed metasurface under oblique incidence, we also calculate the anomalous refraction efficiency (Figures S19–S22, Supplementary material) when 90°/198° linear polarized incident light with different incident angles is transmitted through the designed metasurfaces (the sizes of the (*a*, 1) nanoparticle of the metasurface designed for the case of 90°/198° linear polarized incident light is the same as the sizes of the (1, *b*) nanoparticle in Table S10/S15 respectively). The results show that the designed metasurface has high tolerance for incident angle in the range of (−70° to 20°, the negative sign indicates that the incident angle is deflected along the negative direction of the *x*-axis) under the incident of 90° linear polarized light. However, when the incidence light is 198° linear polarized light, the designed metasurface has an increased sensitivity to the incident angle, but still has a high beam deflection ability for −30° to 0° incident light. However, more cases under oblique incidence still need to be further considered. 4. The equations in Section 3.1 can be used to obtain the theoretical conditions that the parameters (*θ*_(*a*,*b*)_, *θ*_(*c*,*d*)_, *ϕ*_*x*(*a*,*b*)_, *ϕ*_*x*(*c*,*d*)_, *ϕ*_*y*(*a*,*b*)_, *ϕ*_*y*(*c*,*d*)_) need to meet for realizing the maximum polarization beam splitting efficiency. A polarization beam splitting device can also be designed according to the obtained theoretical equations. 5. The conclusions of this study can be extended to reflective metasurfaces, following which the conditions for the establishment of the generalized Snell’s law of reflection in reflective metasurfaces can be obtained.

## 5 Conclusions

This paper first summarizes the expression of the Jones matrix at each position of the metasurface, *J*_(*x*,*y*)_, based on the Jones matrix of the nanopillars at the position (*a*, *b*). We then obtain the (*m*, *n*) term, *J*_(*m*,*n*)_, in the Fourier series of *J*_(*x*,*y*)_. *J*_(*m*,*n*)_ also represents the (*m*, *n*)th transmission coefficient of the transmitted light. Based on *J*_(*m*,*n*)_, we present the anomalous refraction efficiency, *I* ^(*m*,*n*)^, when the incident is transmitted to the (*m*, *n*)th transmission order through the metasurfaces under the incidence of different polarized light. Thereafter, we present the equations that the phase changes introduced by the nanoparticles to the incident light and the rotation angles of the nanoparticles need to meet when *I* ^(*m*,*n*)^ reaches the highest value. In the subsequent design of high-efficiency beam deflection metasurfaces, the parameters related to nanopillars sizes can be selected according to the equations discussed herein.

To prove the accuracy of the derivation process, by using the interior point method, we calculate the phase changes that need to be introduced by the nanopillars to different polarized incident light and the rotation angles of the nanopillars when *I* ^(*m*,*n*)^ reaches the highest value. The calculated results satisfy the derived equations. Moreover, to prove the accuracy of the established theory, we numerically simulate the anomalous refraction efficiency under the incidence of different polarized light when the light is transmitted to the (*m*, *n*)th transmission order through the metasurfaces designed according to the above-derived equations, by using FDTD Solutions. We find that the anomalous refraction efficiency is high and also basically consistent with the theoretical maximum achievable efficiency value. Lastly, we present the conditions for the establishment of the polarization-independent generalized Snell’s law of refraction in all-dielectric metasurfaces.

The results of this study may have potential applications in high efficient beam deflectors [29], structured beam generators [35, 36], polarization detection devices [23], and other fields, and provide theoretical basis for the design of high-efficiency beam deflection metasurfaces.

## Supplementary Material

Supplementary Material
