# Supplementary material for: Conditions for establishing the “generalized Snell’s law of refraction” in all-dielectric metasurfaces: theoretical bases for design of high-efficiency beam deflection metasurfaces
Source: Nanophotonics. 2021 Nov 1;11(1):21–32. doi: 10.1515/nanoph-2021-0459 (PMC11501684; doi:10.1515/nanoph-2021-0459)
Supplement: Supplementary file 1 — Supplementary Material [file j_nanoph-2021-0459_suppl.doc]

Conditions for Establishing the “Generalized Snell’s Law of Refraction” in All-Dielectric Metasurfaces: Theoretical Bases for Design of High-Efficiency Beam Deflection Metasurfaces

Siyuan Shen^a^, Zhaohui Ruan^a^, Yuan Yuan^a,b,*^, Heping Tan^a,b^

^a^ School of Energy Science and Engineering, Harbin Institute of Technology, 92 West Dazhi Street, Harbin 150001, PR China

^b^ Key Laboratory of Aerospace Thermophysics, Ministry of Industry and Information Technology, Harbin Institute of Technology, 92 West Dazhi Street , Harbin 150001, PR China

[**yuanyuan83@hit.edu.cn*](mailto:*yuanyuan83@hit.edu.cn)

**S1. Derivation of the transmission coefficient of (*m*,*n*) order transmitted light**

(S1)

**S2. The conditions that need to be met when the** *I*(*^m,n^*) **reaches the maximum and when the phase variations introduced by each nanopillar in the metasurfaces to different polarized light only depend on the geometric phase under the incidence of circularly polarized light**

When *ϕ_x_*(*_a,b_*) = *ϕ_x_*(*_c,d_*), *ϕ_y_*(*_a,b_*) = *ϕ_y_*(*_c,d_*) in Eq. (19) (or on substituting Eq. (20) and $|q\rangle=\frac{\sqrt{2}}{2}\left[\begin{matrix} 1 \\ \text{i} \end{matrix}\right]$ in Eqs. (6) and (7)), we can obtain：

(S2)

Where$\alpha\text{=}\frac{2\pi}{A}(c-a)m\text{+}\frac{2\pi}{B}(d-b)n$, . When cos*θ* = -1 in Eq. (S2), Eq. (S2) can be further derived to

$\begin{matrix} 2\cos^{2}(\theta_{(a,b)}-\theta_{(c,d)})\cos\alpha+2\sin^{2}(\theta_{(a,b)}-\theta_{(c,d)})\cos\alpha\cos\theta+2\cos(\theta_{(a,b)}-\theta_{(c,d)})\sin(\theta_{(a,b)}-\theta_{(c,d)})\sin\alpha(1-\cos\theta) \\ \text{=}2\cos^{2}(\theta_{(a,b)}-\theta_{(c,d)})\cos\alpha-2\sin^{2}(\theta_{(a,b)}-\theta_{(c,d)})\cos\alpha+4\cos(\theta_{(a,b)}-\theta_{(c,d)})\sin(\theta_{(a,b)}-\theta_{(c,d)})\sin\alpha\\ \text{=2}\cos\alpha\cos(2(\theta_{(a,b)}-\theta_{(c,d)}))+2\sin\alpha\sin(2(\theta_{(a,b)}-\theta_{(c,d)})) \\ =2\cos(2(\theta_{(a,b)}-\theta_{(c,d)})-\alpha). \end{matrix}$ (S3)

When Eq. (S3) is equal to 2 (let Eq. (S3) be equal to 2 is because the maximum value that Eq. (19) can reach is 2), that is when in Eq. (20) and Eq. (21) is satisfied, *I*(*^m,n^*) reaches the maximum.

When *ϕ_x_*(*_a,b_*) = *ϕ_x_*(*_c,d_*), *ϕ_y_*(*_a,b_*) = *ϕ_y_*(*_c,d_*) in Eq. (22) (or on substituting Eq. (20) and $|q\rangle=\frac{\sqrt{2}}{2}\left[\begin{matrix} 1 \\ \text{-i} \end{matrix}\right]$ in Eqs. (6) and (7)), we can obtain：

(S4)

Where$\alpha\text{=}\frac{2\pi}{A}(c-a)m\text{+}\frac{2\pi}{B}(d-b)n$, . When cos*θ* = -1 in Eq. (S4), Eq. (S4) can be further derived to

$\begin{matrix} 2\cos^{2}(\theta_{(a,b)}-\theta_{(c,d)})\cos\alpha+2\sin^{2}(\theta_{(a,b)}-\theta_{(c,d)})\cos\alpha\cos\theta+2\cos(\theta_{(a,b)}-\theta_{(c,d)})\sin(\theta_{(a,b)}-\theta_{(c,d)})\sin\alpha(\cos\theta-1) \\ \text{=}2\cos^{2}(\theta_{(a,b)}-\theta_{(c,d)})\cos\alpha\text{--}2\sin^{2}(\theta_{(a,b)}-\theta_{(c,d)})\cos\alpha-4\cos(\theta_{(a,b)}-\theta_{(c,d)})\sin(\theta_{(a,b)}-\theta_{(c,d)})\sin\alpha\\ \text{=2}\cos\alpha\cos(2(\theta_{(a,b)}-\theta_{(c,d)}))-2\sin\alpha\sin(2(\theta_{(a,b)}-\theta_{(c,d)})) \\ =2\cos(2(\theta_{(a,b)}-\theta_{(c,d)})+\alpha) \end{matrix}$ (S5)

When Eq. (S5) is equal to 2 (let Eq. (S5) be equal to 2 is because the maximum value that Eq. (22) can reach is 2), that is when in Eq. (20) and Eq. (23) is satisfied, *I*(*^m,n^*) reaches the maximum.

**S3. Verification**

We also verify the cases where the nanopillars have rotation angles and the incident light is 30º, 45º, 108º linear polarized light or left-handed circularly polarized light.

(a) 30º linear polarized incident light: The case considered is A = 1, B = 5, (*m*,*n*) = (0,1), and the incident wavelength of 1200 nm. We select suitable nanopillars based on the phase changes calculated according to Eq. (8) (*α* = 30º) by using the interior point method and assign them the calculated rotation angles. The specific sizes of the selected nanopillars, the transmission efficiency through the nanopillars, the calculated phase variations that need to be introduced by the nanopillars, and the actual phase variations introduced by the selected nanopillars are listed in Table S13. Through the simulations in FDTD Solutions, we obtain the anomalous refraction efficiency for the 30º linear polarized incident light transmitted to the (0,1)^th^ order through the metasurface composed by the selected nanopillars (total transmission efficiency T = 0.7322, transmission efficiency projected to the (0,1)^th^ order T(0,1) = 0.6465, and efficiency of anomalous refraction to the (0,1)^th^ order *η* = 0.8829). It is clear that the efficiency is basically the same as the one (*η* = 0.8751) obtained according to Eq. (14). The slightly higher value is likely due to the interference between nanopillars.

(b) 45º linear polarized incident light: The case considered is A = 1, B = 5, (*m*,*n*) = (0,1), and the incident wavelength of 1200 nm. We select suitable nanopillars based on the phase changes calculated according to Eq. (8) (*α* = 45º) by using the interior point method and assign them the calculated rotation angles. The specific sizes of the selected nanopillars, the transmission efficiency through the nanopillars, the calculated phase variations that need to be introduced by the nanopillars, and the actual phase variations introduced by the selected nanopillars are listed in Table S14. Through the simulations in FDTD Solutions, we obtain the anomalous refraction efficiency for the 30º linear polarized incident light transmitted to the (0,1)^th^ order through the metasurface composed by the selected nanopillars (total transmission efficiency T = 0.7599, transmission efficiency projected to the (0,1)^th^ order T(0,1) = 0.6521, and efficiency of anomalous refraction to the (0,1)^th^ order *η* = 0.8581). It is clear that the efficiency is basically the same as the one (*η* = 0.8751) obtained according to Eq. (14). The slightly lower efficiency is likely because the transmission efficiency through the nanopillars is low and the scattering between the nanopillars interferes.

(c) 108º linear polarized incident light: The case considered is A = 1, B = 5, (*m*,*n*) = (0,1), and the incident wavelength of 1200 nm. We select suitable nanopillars based on the phase changes calculated according to Eq. (8) (*α* = 108º) by using the interior point method and assign them the calculated rotation angles. The specific sizes of the selected nanopillars, the transmission efficiency through the nanopillars, the calculated phase variations that need to be introduced by the nanopillars, and the actual phase variations introduced by the selected nanopillars are listed in Table S15. Through the simulations in FDTD Solutions, we obtain the anomalous refraction efficiency for the 45º linear polarized incident light transmitted to the (0,1)^th^ order through the metasurface composed by the selected nanopillars (total transmission efficiency T = 0.565, transmission efficiency projected to the (0,1)^th^ order T(0,1) = 0.4821, and efficiency of anomalous refraction to the (0,1)^th^ order *η* = 0.8531). It is clear that the efficiency is basically the same as the one (*η* = 0.8751) obtained according to Eq. (14). The slightly lower efficiency is likely because the transmission efficiency through the nanopillars is low and the scattering between the nanopillars interferes.

We also provide the simulated corresponding electric field component field patterns, far-field electric field intensity as a function of the diffraction angle and far field electric field distribution respectively (Figure S12-S14, Supplementary Material) under the incidence of 30º, 45º, 108º linear polarized incident light. The results prove the high anomalous refraction efficiency of different polarized incident light transmitted to the (0,1)^th^ order through the metasurfaces, and the deflection angle estimated (zenith angle (*θ*) = 28.5º) in simulation (Figure S13) is also consistent with the theoretical value of which is derived from the generalized Snell’s law (*θ* = arcsin(*λ* / B / *p_y_*) * 180 / π= 28.7º).

(d) Left-handed circularly polarized light: The case considered is A = 1, B = 5, (*m*,*n*) = (0,1), and the incident wavelength of 1190 nm. We select suitable nanopillars based on the phase changes calculated according to Eq. (19) by using the interior point method and assign them the calculated rotation angles. The specific sizes of the selected nanopillars, the transmission efficiency through the nanopillars, the calculated phase variations that need to be introduced by the nanopillars, and the actual phase variations introduced by the selected nanopillars are presented in Table S16. Through the simulations in FDTD Solutions, we obtain the anomalous refraction efficiency for the left-handed circularly polarized incident light transmitted to the (0,1)^th^ order through the metasurface composed by the selected nanopillars (total transmission efficiency T = 0.6974, transmission efficiency projected to the (0,1)^th^ order T(0,1) = 0.5967, and efficiency of anomalous refraction to the (0,1)^th^ order *η* = 0.8556). Based on the results, we noted that the efficiency is also basically the same as the one (*η* = 0.8751) obtained according to Eq. (14). The slightly lower efficiency is likely because the transmission efficiency through the nanopillars is low and the scattering between the nanopillars interferes. We also provide the far-field electric field intensity as a function of the diffraction angle and far field electric field distribution respectively (Figure S15-S16, Supplementary Material). The results prove the high anomalous refraction efficiency of left-handed circularly polarized incident light transmitted to the (0,1)^th^ order through the metasurfaces, and the deflection angle estimated (zenith angle (*θ*) = 27.6º) in simulation (Figure S15) is also consistent with the theoretical value of which is derived from the generalized Snell’s law (*θ* = arcsin(*λ* / B / *p_y_*) * 180 / π= 28.4º).

Table S1 The phase changes that need to be introduced by the nanopillars to the incident light and the rotation angles of the nanopillars when *I*(*^m,n^*) reaches the maximum value under the incidence of different polarized light calculated by the interior point method when A = 1 and B = 5 and (*m*,*n*) = (0,1).

| the phase changes that need to be introduced and the rotation angles | 0º linear polarized light | 30º linear polarized light | 45º linear polarized light | | 108º linear polarized light | left-handed circularly polarized light | right-handed circularly polarized light |
| --- | --- | --- | --- | --- | --- | --- | --- |
| *ϕ_x_*_(1,1)_ | 1.851883 | 1.942652 | 1.729332 | 0.759974 | | 1.938493 | 0.591995 |
| *ϕ_x_*_(1,2)_ | 3.103521 | 3.199319 | 1.920349 | 2.016611 | | 3.195129 | 1.848629 |
| *ϕ_x_*_(1,3)_ | 4.362346 | 4.456082 | 3.176978 | 3.273248 | | 4.451766 | 3.105265 |
| *ϕ_x_*_(1,4)_ | 5.618984 | 4.933509 | 4.433618 | 4.529885 | | 4.301682 | 4.361901 |
| *ϕ_x_*_(1,5)_ | 1.858171 | 0.686357 | 5.690254 | 3.907109 | | 0.681855 | 5.618537 |
| *ϕ_y_*_(1,1)_ | 1.848838 | 1.943027 | 0.663705 | 0.759982 | | 0.531772 | 0.591998 |
| *ϕ_y_*_(1,2)_ | 3.106012 | 3.199668 | 1.92034 | 2.016626 | | 1.788406 | 1.848631 |
| *ϕ_y_*_(1,3)_ | 2.928136 | 4.456329 | 3.176963 | 3.273261 | | 3.045044 | 3.105268 |
| *ϕ_y_*_(1,4)_ | 3.944432 | 5.712907 | 4.173406 | 4.02746 | | 5.708402 | 4.361904 |
| *ϕ_y_*_(1,5)_ | 0.592436 | 0.829605 | 2.513872 | 5.786523 | | 5.55832 | 5.61854 |
| *θ*_(1,1)_ | 1.850049 | 1.340648 | 0.785398 | 1.094359 | | 1.064651 | 1.422078 |
| *θ*_(1,2)_ | 1.22173 | 1.364229 | 1.204277 | 1.075415 | | 1.064651 | 1.407639 |
| *θ*_(1,3)_ | 3.141593 | 1.549891 | 2.164208 | 1.116125 | | 1.064651 | 1.686392 |
| *θ*_(1,4)_ | 3.141593 | 1.047328 | 2.356194 | 0.785394 | | 2.635447 | 1.394151 |
| *θ*_(1,5)_ | 1.570796 | 2.617173 | 2.356194 | 2.356193 | | 1.064651 | 1.324059 |

Table S2 The highest achievable efficiency of anomalous refraction to (1,1)^th^ order under the incidence of different polarized light calculated by the interior point method when A = 2 and B = 2, A = 3 and B = 3, A = 4 and B = 4, A = 5 and B = 5 or A = 6 and B = 6 respectively.

| the polarization states of the incident light | the highest achievable efficiency when A = 2, B = 2 | the highest achievable efficiency when A = 3, B = 3 | the highest achievable efficiency when A = 4, B = 4 | the highest achievable efficiency when A = 5, B = 5 | | the highest achievable efficiency when A = 6, B = 6 |
| --- | --- | --- | --- | --- | --- | --- |
| 0º linear polarized light | 0.1643 | 0.4677 | 0.6544 | 0.7644 | 0.8277 | |
| 90º linear polarized light | 0.1643 | 0.4677 | 0.6539 | 0.7646 | 0.8277 | |
| 45º linear polarized light | 0.1643 | 0.4677 | 0.6558 | 0.7653 | 0.8271 | |
| 135º linear polarized light | 0.1643 | 0.4677 | 0.6566 | 0.7632 | 0.8271 | |
| left-handed circularly polarized light | 0.1643 | 0.4677 | 0.6545 | 0.7645 | 0.8294 | |
| right-handed circularly polarized light | 0.1643 | 0.4677 | 0.6569 | 0.7645 | 0.8294 | |

Table S3 The phase changes that need to be introduced by the nanopillars to the incident light when *I*(*^m,n^*) reaches the maximum value under the incidence of different polarized light calculated by the interior point method when A = 5 and B = 5, (*m*,*n*) = (1,1) and the nanopillars in the metasurfaces have no rotation angles.

| the phase changes that need to be introduced | 0º linear polarized light | 90º linear polarized light | 45º linear polarized light | 135º linear polarized light | left-handed circularly polarized light | right-handed circularly polarized light |
| --- | --- | --- | --- | --- | --- | --- |
| *ϕ_x_*_(1,1)_ | 1.254578 | 2.176152 | 1.24977 | 1.24977 | 1.24977 | 1.24977 |
| *ϕ_x_*_(1,2)_ | 2.511198 | 2.176152 | 2.505655 | 2.505655 | 2.505655 | 2.505655 |
| *ϕ_x_*_(1,3)_ | 3.767844 | 3.019452 | 3.763029 | 3.763029 | 3.763029 | 3.763029 |
| *ϕ_x_*_(1,4)_ | 5.024524 | 3.019452 | 5.020671 | 5.020671 | 5.020671 | 5.020671 |
| *ϕ_x_*_(1,5)_ | 0.004184 | 2.176152 | 0.013548 | 0.013548 | 0.013548 | 0.013548 |
| *ϕ_x_*_(2,1)_ | 2.511219 | 3.019452 | 2.506835 | 2.506835 | 2.506835 | 2.506835 |
| *ϕ_x_*_(2,2)_ | 3.767872 | 2.176152 | 3.762826 | 3.762826 | 3.762826 | 3.762826 |
| *ϕ_x_*_(2,3)_ | 5.024496 | 1.471917 | 5.018733 | 5.018733 | 5.018733 | 5.018733 |
| *ϕ_x_*_(2,4)_ | 6.276984 | 3.019452 | 6.262617 | 6.262617 | 6.262617 | 6.262617 |
| *ϕ_x_*_(2,5)_ | 1.254578 | 2.176152 | 1.24977 | 1.24977 | 1.24977 | 1.24977 |
| *ϕ_x_*_(3,1)_ | 3.76785 | 1.471917 | 3.761789 | 3.761789 | 3.761789 | 3.761789 |
| *ϕ_x_*_(3,2)_ | 5.024482 | 2.176152 | 5.019181 | 5.019181 | 5.019181 | 5.019181 |
| *ϕ_x_*_(3,3)_ | 0.004188 | 1.471917 | 0.013619 | 0.013619 | 0.013619 | 0.013619 |
| *ϕ_x_*_(3,4)_ | 1.25462 | 3.019452 | 1.249412 | 1.249412 | 1.249412 | 1.249412 |
| *ϕ_x_*_(3,5)_ | 2.511198 | 2.176152 | 2.505655 | 2.505655 | 2.505655 | 2.505655 |
| *ϕ_x_*_(4,1)_ | 5.024482 | 2.176152 | 5.019181 | 5.019181 | 5.019181 | 5.019181 |
| *ϕ_x_*_(4,2)_ | 6.276828 | 3.874334 | 6.26212 | 6.26212 | 6.26212 | 6.26212 |
| *ϕ_x_*_(4,3)_ | 1.25462 | 3.019452 | 1.249412 | 1.249412 | 1.249412 | 1.249412 |
| *ϕ_x_*_(4,4)_ | 2.511218 | 3.019452 | 2.506835 | 2.506835 | 2.506835 | 2.506835 |
| *ϕ_x_*_(4,5)_ | 3.767872 | 2.176152 | 3.762826 | 3.762826 | 3.762826 | 3.762826 |
| *ϕ_x_*_(5,1)_ | 6.277008 | 4.64191 | 6.262558 | 6.262558 | 6.262558 | 6.262558 |
| *ϕ_x_*_(5,2)_ | 1.254578 | 2.176152 | 1.24977 | 1.24977 | 1.24977 | 1.24977 |
| *ϕ_x_*_(5,3)_ | 2.511231 | 1.471917 | 2.505601 | 2.505601 | 2.505601 | 2.505601 |
| *ϕ_x_*_(5,4)_ | 3.767844 | 3.019452 | 3.763029 | 3.763029 | 3.763029 | 3.763029 |
| *ϕ_x_*_(5,5)_ | 5.024524 | 3.019452 | 5.020671 | 5.020671 | 5.020671 | 5.020671 |
| *ϕ_y_*_(1,1)_ | 2.176153 | 1.254578 | 1.24977 | 1.24977 | 1.24977 | 1.24977 |
| *ϕ_y_*_(1,2)_ | 2.176153 | 2.511197 | 2.505656 | 2.505656 | 2.505656 | 2.505656 |
| *ϕ_y_*_(1,3)_ | 3.019452 | 3.767843 | 3.76303 | 3.76303 | 3.76303 | 3.76303 |
| *ϕ_y_*_(1,4)_ | 3.019452 | 5.024524 | 5.020671 | 5.020671 | 5.020671 | 5.020671 |
| *ϕ_y_*_(1,5)_ | 2.176153 | 0.004184 | 0.013555 | 0.013555 | 0.013555 | 0.013555 |
| *ϕ_y_*_(2,1)_ | 3.019452 | 2.511218 | 2.506835 | 2.506835 | 2.506835 | 2.506835 |
| *ϕ_y_*_(2,2)_ | 2.176153 | 3.767871 | 3.762826 | 3.762826 | 3.762826 | 3.762826 |
| *ϕ_y_*_(2,3)_ | 1.471918 | 5.024495 | 5.018733 | 5.018733 | 5.018733 | 5.018733 |
| *ϕ_y_*_(2,4)_ | 3.019452 | 6.276983 | 6.262617 | 6.262617 | 6.262617 | 6.262617 |
| *ϕ_y_*_(2,5)_ | 2.176153 | 1.254578 | 1.24977 | 1.24977 | 1.24977 | 1.24977 |
| *ϕ_y_*_(3,1)_ | 1.471918 | 3.76785 | 3.76179 | 3.76179 | 3.76179 | 3.76179 |
| *ϕ_y_*_(3,2)_ | 2.176153 | 5.024481 | 5.019181 | 5.019181 | 5.019181 | 5.019181 |
| *ϕ_y_*_(3,3)_ | 1.471918 | 0.004188 | 0.013618 | 0.013618 | 0.013618 | 0.013618 |
| *ϕ_y_*_(3,4)_ | 3.019452 | 1.254619 | 1.249413 | 1.249413 | 1.249413 | 1.249413 |
| *ϕ_y_*_(3,5)_ | 2.176153 | 2.511197 | 2.505656 | 2.505656 | 2.505656 | 2.505656 |
| *ϕ_y_*_(4,1)_ | 2.176153 | 5.024481 | 5.019181 | 5.019181 | 5.019181 | 5.019181 |
| *ϕ_y_*_(4,2)_ | 3.874333 | 6.276828 | 6.26212 | 6.26212 | 6.26212 | 6.26212 |
| *ϕ_y_*_(4,3)_ | 3.019452 | 1.254619 | 1.249413 | 1.249413 | 1.249413 | 1.249413 |
| *ϕ_y_*_(4,4)_ | 3.019452 | 2.511218 | 2.506835 | 2.506835 | 2.506835 | 2.506835 |
| *ϕ_y_*_(4,5)_ | 2.176153 | 3.767871 | 3.762826 | 3.762826 | 3.762826 | 3.762826 |
| *ϕ_y_*_(5,1)_ | 4.64191 | 6.277007 | 6.262559 | 6.262559 | 6.262559 | 6.262559 |
| *ϕ_y_*_(5,2)_ | 2.176153 | 1.254578 | 1.24977 | 1.24977 | 1.24977 | 1.24977 |
| *ϕ_y_*_(5,3)_ | 1.471918 | 2.51123 | 2.505601 | 2.505601 | 2.505601 | 2.505601 |
| *ϕ_y_*_(5,4)_ | 3.019452 | 3.767843 | 3.76303 | 3.76303 | 3.76303 | 3.76303 |
| *ϕ_y_*_(5,5)_ | 3.019452 | 5.024524 | 5.020671 | 5.020671 | 5.020671 | 5.020671 |

Table S4 The phase changes that need to be introduced by the nanopillars to the incident light and the rotation angles of the nanopillars when *I*(*^m,n^*) reaches the maximum value under the incidence of different circularly polarized light calculated by the interior point method when A = 1 and B = 5, (*m*,*n*) = (0,1) and only changing the rotation angles of the nanopillars.

| the phase changes that need to be introduced and the rotation angles | left-handed circularly polarized light | right-handed circularly polarized light |
| --- | --- | --- |
| *ϕ_x_* | 3.14 | 1.462488 |
| *ϕ_y_* | 6.28 | 4.604081 |
| *θ*_(1,1)_ | 0.311963 | 0.93811 |
| *θ*_(1,2)_ | 2.825235 | 1.566428 |
| *θ*_(1,3)_ | 2.196917 | 2.194746 |
| *θ*_(1,4)_ | 1.568599 | 2.823066 |
| *θ*_(1,5)_ | 0.94028 | 0.309792 |

Table S5 The sizes of the selected nanopillars, the transmission efficiency through the nanopillars under the incidence of 0º linear polarized light, and the actual phase variations introduced by the selected nanopillars when A = 5 and B = 5, (*m*,*n*) = (1,1), and the incident wavelength is 1300 nm.

| the nanopillar position | the side length *d_x_* / *d_y_* (nm) | *ϕ_x_* | *t_x_* |
| --- | --- | --- | --- |
| (1,1) | 370 | 1.25998 | 0.912867 |
| (1,2) | 70 | 2.50553 | 0.876426 |
| (1,3) | 235 | 3.76279 | 0.8316 |
| (1,4) | 275 | 5 | 0.728346 |
| (1,5) | 310 | 6.20813 | 0.684644 |
| (2,1) | 70 | 2.50553 | 0.876426 |
| (2,2) | 235 | 3.76279 | 0.8316 |
| (2,3) | 275 | 5 | 0.728346 |
| (2,4) | 310 | 6.20813 | 0.684644 |
| (2,5) | 370 | 1.25998 | 0.912867 |
| (3,1) | 235 | 3.76279 | 0.8316 |
| (3,2) | 275 | 5 | 0.728346 |
| (3,3) | 310 | 6.20813 | 0.684644 |
| (3,4) | 370 | 1.25998 | 0.912867 |
| (3,5) | 70 | 2.50553 | 0.876426 |
| (4,1) | 275 | 5 | 0.728346 |
| (4,2) | 310 | 6.20813 | 0.684644 |
| (4,3) | 370 | 1.25998 | 0.912867 |
| (4,4) | 70 | 2.50553 | 0.876426 |
| (4,5) | 235 | 3.76279 | 0.8316 |
| (5,1) | 310 | 6.20813 | 0.684644 |
| (5,2) | 370 | 1.25998 | 0.912867 |
| (5,3) | 70 | 2.50553 | 0.876426 |
| (5,4) | 235 | 3.76279 | 0.8316 |
| (5,5) | 275 | 5 | 0.728346 |

Table S6 The simulation results of the total transmission efficiency, the transmission efficiency projected to the (1,1)^th^ order, and the efficiency of anomalous refraction to the (1,1)^th^ order of light passing through the metasurfaces composed by the selected nanopillars under the incidence of different polarized light by FDTD when A = 5 and B = 5, (*m*,*n*) = (1,1), the incident wavelength is 1300 nm, and the nanopillars have no rotation angles.

| the polarization states of the incident light | the total  transmission efficiency  T | the transmission efficiency projected to the (1,1)^th^ order T(1,1) | the efficiency of anomalous refraction to the (1,1)^th^ order  *η* |
| --- | --- | --- | --- |
| 0º linear polarized light | 0.7171 | 0.5553 | 0.7744 |
| 90º linear polarized light | 0.727 | 0.57 | 0.784 |
| 45º linear polarized light | 0.7526 | 0.6453 | 0.8575 |
| 135º linear polarized light | 0.7012 | 0.4939 | 0.7043 |
| left-handed circularly polarized light | 0.7269 | 0.57 | 0.7842 |
| right-handed circularly polarized light | 0.7269 | 0.5692 | 0.783 |

Table S7 The sizes of the nanopillars selected based on the phase data calculated according to Eq. (13), the transmission efficiency of the incident light through the nanopillars, the calculated phase variations that need to be introduced by the nanopillars, and the actual phase variations introduced by the selected nanopillars when A = 1 and B = 5, (*m*,*n*) = (0,1), the incident light is 1300 nm 0º linear polarized light, and the nanopillars have no rotation angles.

| the nanopillar position | | the x direction side  length *d_x_* (nm) | the y direction side length *d_y_* (nm) | *ϕ_x_^actual^* | *ϕ_x_* | *t_x_^actual^* | *t_x_* |
| --- | --- | --- | --- | --- | --- | --- | --- |
| (1,1) | 320 | | 400 | 0.783585 | 0.783585 | 0.863031 | 1 |
| (1,2) | 410 | | 400 | 2.064035 | 2.04022 | 0.794726 | 1 |
| (1,3) | 210 | | 210 | 3.294185 | 3.29686 | 0.846567 | 1 |
| (1,4) | 230 | | 380 | 4.552235 | 4.553496 | 0.833951 | 1 |
| (1,5) | 260 | | 420 | 5.818927 | 5.81013 | 0.749943 | 1 |

Table S8 The sizes of the nanopillars selected based on the phase values linearly selected from 0-2π, the transmission efficiency of the incident light through the nanopillars, the phase variations that need to be introduced by the nanopillars, and the actual phase variations introduced by the selected nanopillars when A = 1 and B = 5, (*m*,*n*) = (0,1), the incident light is 1300 nm 0º linear polarized light, and the nanopillars have no rotation angles.

| the nanopillar position | the x direction side length *d_x_* (nm) | the y direction side length *d_y_* (nm) | *ϕ_x_^actual^* | *ϕ_x_* | *t_x_^actual^* | *t_x_* |
| --- | --- | --- | --- | --- | --- | --- |
| (1,1) | 290 | 370 | 0.00717 | 0 | 0.719773 | 1 |
| (1,2) | 370 | 420 | 1.57966 | 1.5708 | 0.952566 | 1 |
| (1,3) | 220 | 150 | 3.149695 | 3.14159 | 0.857362 | 1 |
| (1,4) | 240 | 360 | 4.72068 | 4.71239 | 0.814453 | 1 |
| (1,5) | 330 | 280 | 6.27608 | 6.28318 | 0.650671 | 1 |

Table S9 The simulation results of the total transmission efficiency, the transmission efficiency projected to the (0,1)^th^ order, and the efficiency of anomalous refraction to the (0,1)^th^ order of light passing through the two metasurfaces designed by different methods by FDTD when A = 1 and B = 5, (*m*,*n*) = (0,1), the incident light is 1300 nm 0º linear polarized light, and the nanopillars have no rotation angles.

| design method | the total  transmission efficiency  T | the transmission efficiency projected to the (0,1)^th^ order T(0,1) | the efficiency of anomalous refraction to the (0,1)^th^ order  *η* |
| --- | --- | --- | --- |
| Eq. (13) | 0.713048 | 0.696873 | 0.9773 |
| linearly selected from 0-2π | 0.7703 | 0.5758 | 0.7475 |

Table S10 The sizes of the selected nanopillars, the transmission efficiency of the incident light through the nanopillars, the calculated phase variations that need to be introduced by the nanopillars, and the actual phase variations introduced by the selected nanopillars when A = 1 and B = 5, (*m*,*n*) = (0,1), the incident light is 1300 nm 0º linear polarized light, and the nanopillars have rotation angles.

| The nanopillar position | (1,1) | (1,2) | (1,3) | (1,4) | (1,5) |
| --- | --- | --- | --- | --- | --- |
| the x direction side length *d_x_* (nm) | 400 | 190 | 340 | 330 | 440 |
| the y direction side length *d_y_*(nm) | 400 | 190 | 140 | 220 | 290 |
| rotation angle (º) | 106 | 70 | 180 | 180 | 90 |
| *ϕ_x_^actual^* | 1.824745 | 3.075545 | 4.278265 | 5.517707 | 1.37374 |
| *ϕ_x_* | 1.851883 | 3.103521 | 4.362346 | 5.618984 | 1.858171 |
| *t_x_^actual^* | 0.910273 | 0.857368 | 0.814556 | 0.604164 | 0.858102 |
| *t_x_* | 1 | 1 | 1 | 1 | 1 |
| *ϕ_y_^actual^* | 1.824745 | 3.075545 | 2.989915 | 3.994815 | 0.531335 |
| *ϕ_y_* | 1.848838 | 3.106012 | 2.928136 | 3.944432 | 0.592436 |
| *t_y_^actual^* | 0.910273 | 0.857368 | 0.86322 | 0.832788 | 0.822897 |
| *t_y_* | 1 | 1 | 1 | 1 | 1 |

Table S11 The selected uniform size of the nanopillars, the transmission efficiency of the incident light through the nanopillars, the calculated phase variations that need to be introduced by the nanopillars, and the actual phase variations introduced by the selected nanopillars when A = 1 and B = 5, (*m*,*n*) = (0,1), the incident light is 1200 nm left-handed circularly polarized light, and when the size of the nanopillars of the metasurface is unchanged and only the rotation angles are changed.

| the x direction side length *d_x_*(nm) | the y direction side length *d_y_*(nm) | *ϕ_x_^actual^* | *ϕ_x_* | *t_x_^actual^* | *ϕ_y_^actual^* | *ϕ_y_* | *t_y_^actual^* |
| --- | --- | --- | --- | --- | --- | --- | --- |
| 120 | 400 | 3.169485 | 3.14 | 0.870255 | 5.5687 | 6.28 | 0.669165 |

Table S12 The rotation angles of the nanopillars at different positions calculated according to Eq. (21) by using the interior point method when A = 1 and B = 5, (*m*,*n*) = (0,1), and the incident light is 1200 nm left-handed circularly polarized light.

| the nanopillar position | The rotation angle |
| --- | --- |
| (1,1) | 2.827433 |
| (1,2) | 0.314159 |
| (1,3) | 0.942478 |
| (1,4) | 1.570796 |
| (1,5) | 2.199115 |

Table S13 The sizes of the selected nanopillars, the transmission efficiency of the incident light through the nanopillars, the calculated phase variations that need to be introduced by the nanopillars, and the actual phase variations introduced by the selected nanopillars when A = 1 and B = 5, (*m*,*n*) = (0,1), the incident light is 1200 nm 30º linear polarized light, and the nanopillars have rotation angles.

| The nanopillar position | (1,1) | (1,2) | (1,3) | (1,4) | (1,5) |
| --- | --- | --- | --- | --- | --- |
| the x direction side length *d_x_* (nm) | 360 | 330 | 170 | 230 | 400 |
| the y direction side length *d_y_*(nm) | 260 | 330 | 170 | 220 | 100 |
| rotation angle (º) | 45 | 69 | 124 | 135 | 135 |
| *ϕ_x_^actual^* | 1.742515 | 1.939135 | 3.179845 | 4.385015 | 4.911465 |
| *ϕ_x_* | 1.729332 | 1.920349 | 3.176978 | 4.433618 | 5.690254 |
| *t_x_^actual^* | 0.835946 | 0.866511 | 0.872006 | 0.894147 | 0.852361 |
| *t_x_* | 1 | 1 | 1 | 1 | 1 |
| *ϕ_y_^actual^* | 0.674795 | 1.939135 | 3.179845 | 4.17506 | 3.026985 |
| *ϕ_y_* | 0.663705 | 1.92034 | 3.176963 | 4.173406 | 2.513872 |
| *t_y_^actual^* | 0.781943 | 0.866511 | 0.872006 | 0.902729 | 0.869813 |
| *t_y_* | 1 | 1 | 1 | 1 | 1 |

Table S14 The sizes of the selected nanopillars, the transmission efficiency of the incident light through the nanopillars, the calculated phase variations that need to be introduced by the nanopillars, and the actual phase variations introduced by the selected nanopillars when A = 1 and B = 5, (*m*,*n*) = (0,1), the incident light is 1200 nm 45º linear polarized light, and the nanopillars have rotation angles.

| The nanopillar position | (1,1) | (1,2) | (1,3) | (1,4) | (1,5) |
| --- | --- | --- | --- | --- | --- |
| the x direction side length *d_x_* (nm) | 360 | 330 | 170 | 230 | 400 |
| the y direction side length *d_y_*(nm) | 260 | 330 | 170 | 220 | 100 |
| rotation angle (º) | 45 | 69 | 124 | 135 | 135 |
| *ϕ_x_^actual^* | 1.742515 | 1.939135 | 3.179845 | 4.385015 | 4.911465 |
| *ϕ_x_* | 1.729332 | 1.920349 | 3.176978 | 4.433618 | 5.690254 |
| *t_x_^actual^* | 0.835946 | 0.866511 | 0.872006 | 0.894147 | 0.852361 |
| *t_x_* | 1 | 1 | 1 | 1 | 1 |
| *ϕ_y_^actual^* | 0.674795 | 1.939135 | 3.179845 | 4.17506 | 3.026985 |
| *ϕ_y_* | 0.663705 | 1.92034 | 3.176963 | 4.173406 | 2.513872 |
| *t_y_^actual^* | 0.781943 | 0.866511 | 0.872006 | 0.902729 | 0.869813 |
| *t_y_* | 1 | 1 | 1 | 1 | 1 |

Table S15 The sizes of the selected nanopillars, the transmission efficiency of the incident light through the nanopillars, the calculated phase variations that need to be introduced by the nanopillars, and the actual phase variations introduced by the selected nanopillars when A = 1 and B = 5, (*m*,*n*) = (0,1), the incident light is 1200 nm 108º linear polarized light, and the nanopillars have rotation angles.

| The nanopillar position | (1,1) | (1,2) | (1,3) | (1,4) | (1,5) |
| --- | --- | --- | --- | --- | --- |
| the x direction side length *d_x_* (nm) | 360 | 330 | 170 | 230 | 400 |
| the y direction side length *d_y_*(nm) | 260 | 330 | 170 | 220 | 100 |
| rotation angle (º) | 45 | 69 | 124 | 135 | 135 |
| *ϕ_x_^actual^* | 1.742515 | 1.939135 | 3.179845 | 4.385015 | 4.911465 |
| *ϕ_x_* | 1.729332 | 1.920349 | 3.176978 | 4.433618 | 5.690254 |
| *t_x_^actual^* | 0.835946 | 0.866511 | 0.872006 | 0.894147 | 0.852361 |
| *t_x_* | 1 | 1 | 1 | 1 | 1 |
| *ϕ_y_^actual^* | 0.674795 | 1.939135 | 3.179845 | 4.17506 | 3.026985 |
| *ϕ_y_* | 0.663705 | 1.92034 | 3.176963 | 4.173406 | 2.513872 |
| *t_y_^actual^* | 0.781943 | 0.866511 | 0.872006 | 0.902729 | 0.869813 |
| *t_y_* | 1 | 1 | 1 | 1 | 1 |

Table S16 The sizes of the selected nanopillars, the transmission efficiency of the incident light through the nanopillars, the calculated phase variations that need to be introduced by the nanopillars, and the actual phase variations introduced by the selected nanopillars when A = 1 and B = 5, (*m*,*n*) = (0,1), the incident light is 1190 nm left-handed circularly polarized light, and the nanopillars have rotation angles.

| The nanopillar position | (1,1) | (1,2) | (1,3) | (1,4) | (1,5) |
| --- | --- | --- | --- | --- | --- |
| the x direction side length *d_x_* (nm) | 370 | 380 | 320 | 210 | 320 |
| the y direction side length *d_y_*(nm) | 260 | 310 | 120 | 270 | 230 |
| rotation angle (º) | 61 | 61 | 61 | 151 | 61 |
| *ϕ_x_^actual^* | 2.074195 | 2.724435 | 4.666345 | 4.357185 | 0.916443 |
| *ϕ_x_* | 1.938493 | 3.195129 | 4.451766 | 4.301682 | 0.681855 |
| *t_x_^actual^* | 0.76878 | 0.547941 | 0.84371 | 0.924136 | 0.696182 |
| *t_x_* | 1 | 1 | 1 | 1 | 1 |
| *ϕ_y_^actual^* | 0.926327 | 2.028335 | 3.115615 | 5.700515 | 5.67943 |
| *ϕ_y_* | 0.531772 | 1.788406 | 3.045044 | 5.708402 | 5.55832 |
| *t_y_^actual^* | 0.831073 | 0.895048 | 0.871379 | 0.61368 | 0.760222 |
| *t_y_* | 1 | 1 | 1 | 1 | 1 |


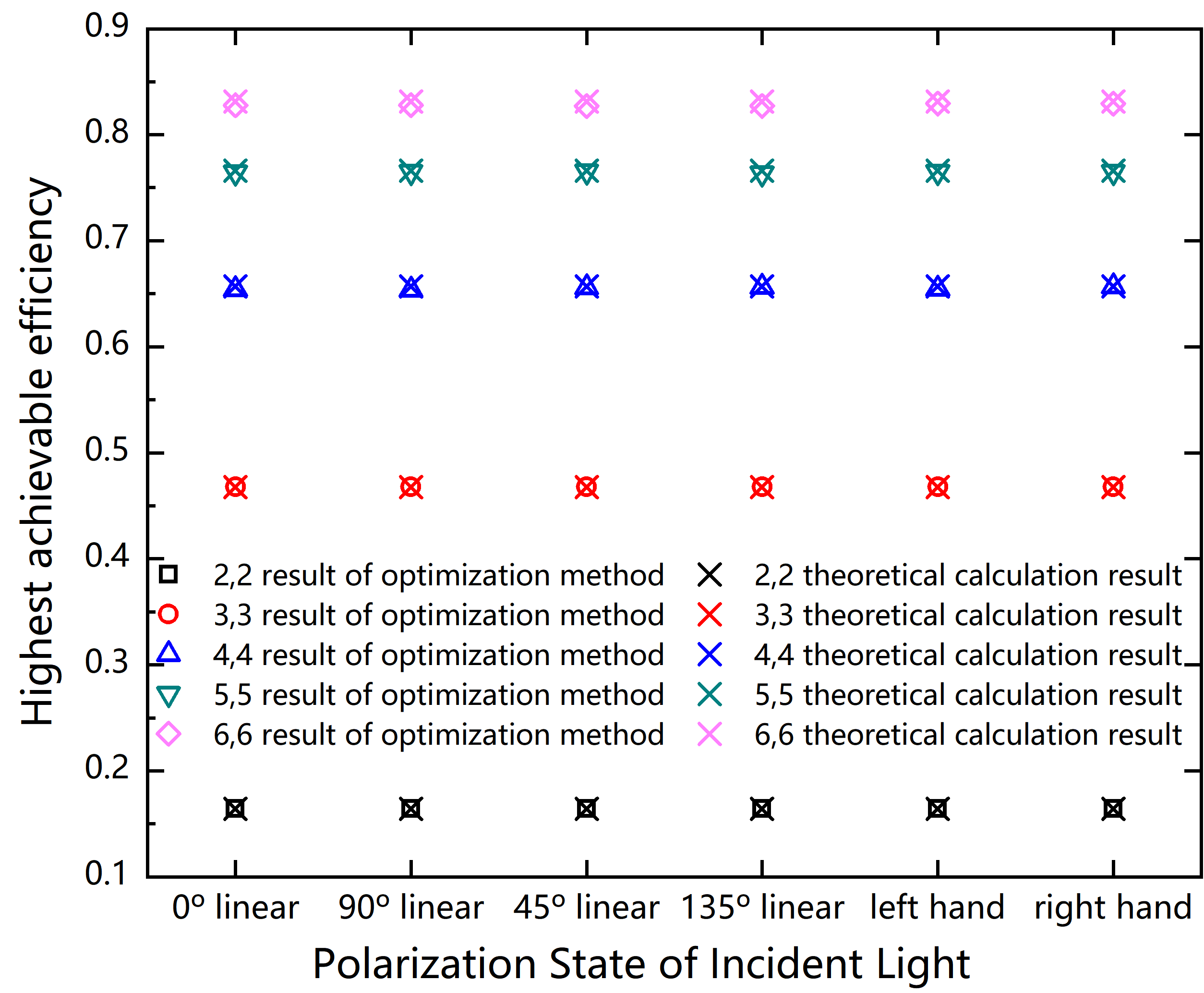


Fig. S1 Comparison between the highest achievable efficiency of anomalous refraction to (1,1)^th^ order under the incidence of different polarized light calculated by the interior point method and the theoretically achievable maximum efficiency obtained according to Eq. (9) when A = 2 and B = 2, A = 3 and B = 3, A = 4 and B = 4, A = 5 and B = 5 or A = 6 and B = 6 respectively.


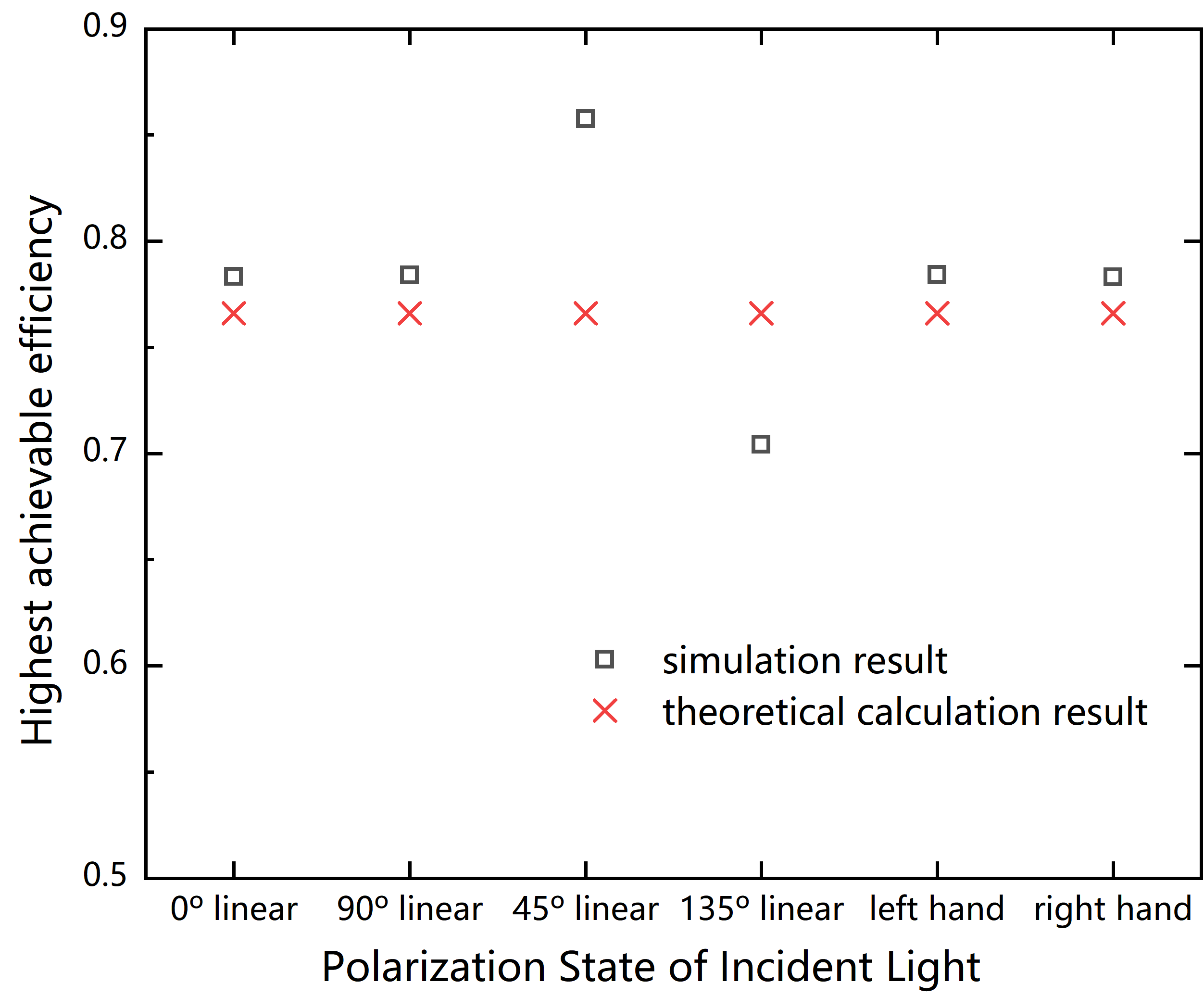


Fig. S2 Comparison between the anomalous refraction efficiency of the incident light transmitted to the (1,1)^th^ order through the metasurfaces composed of the selected nanopillars under the incidence of different polarized light obtained by FDTD simulations and the theoretically achievable maximum efficiency obtained according to Eq. (9) when A = 5 and B = 5, (*m*,*n*) = (1,1), and the incident wavelength is 1300 nm.


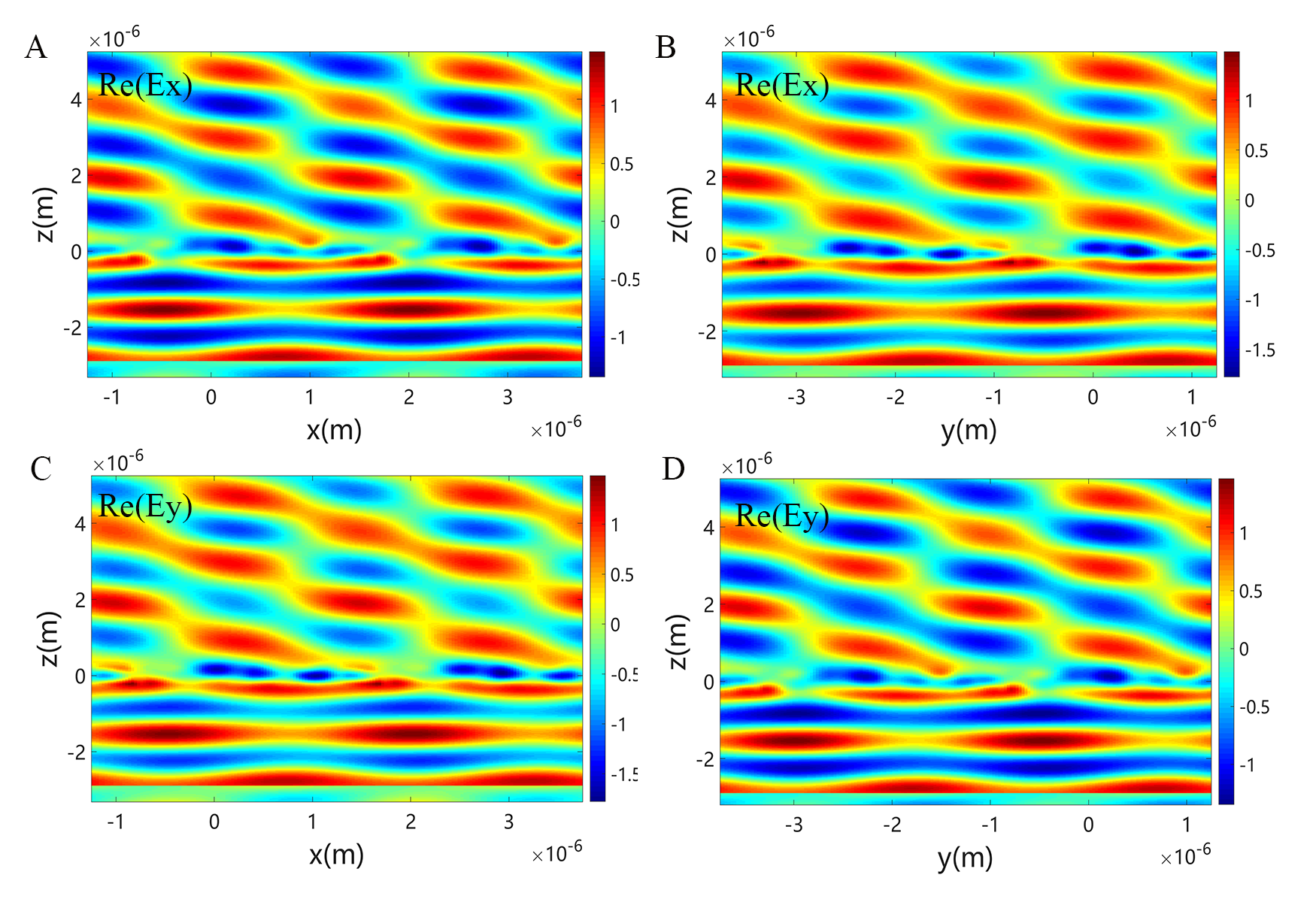


Fig. S3 Corresponding electric field component field patterns in the xz/yz plane under the incidence of 0º linear and 90º linear polarized light when A = 5 and B = 5, (*m*,*n*) = (1,1), the incident wavelength is 1300 nm, and the nanopillars have rotation angles.

(A) Ex component of the transmission wave in the xz plane under the incidence of 0º linear polarized light. (B) Ex component of the transmission wave in the yz plane under the incidence of 0º linear polarized light. (C) Ey component of the transmission wave in the xz plane under the incidence of 90º linear polarized light. (D) Ey component of the transmission wave in the yz plane under the incidence of 90º linear polarized light.


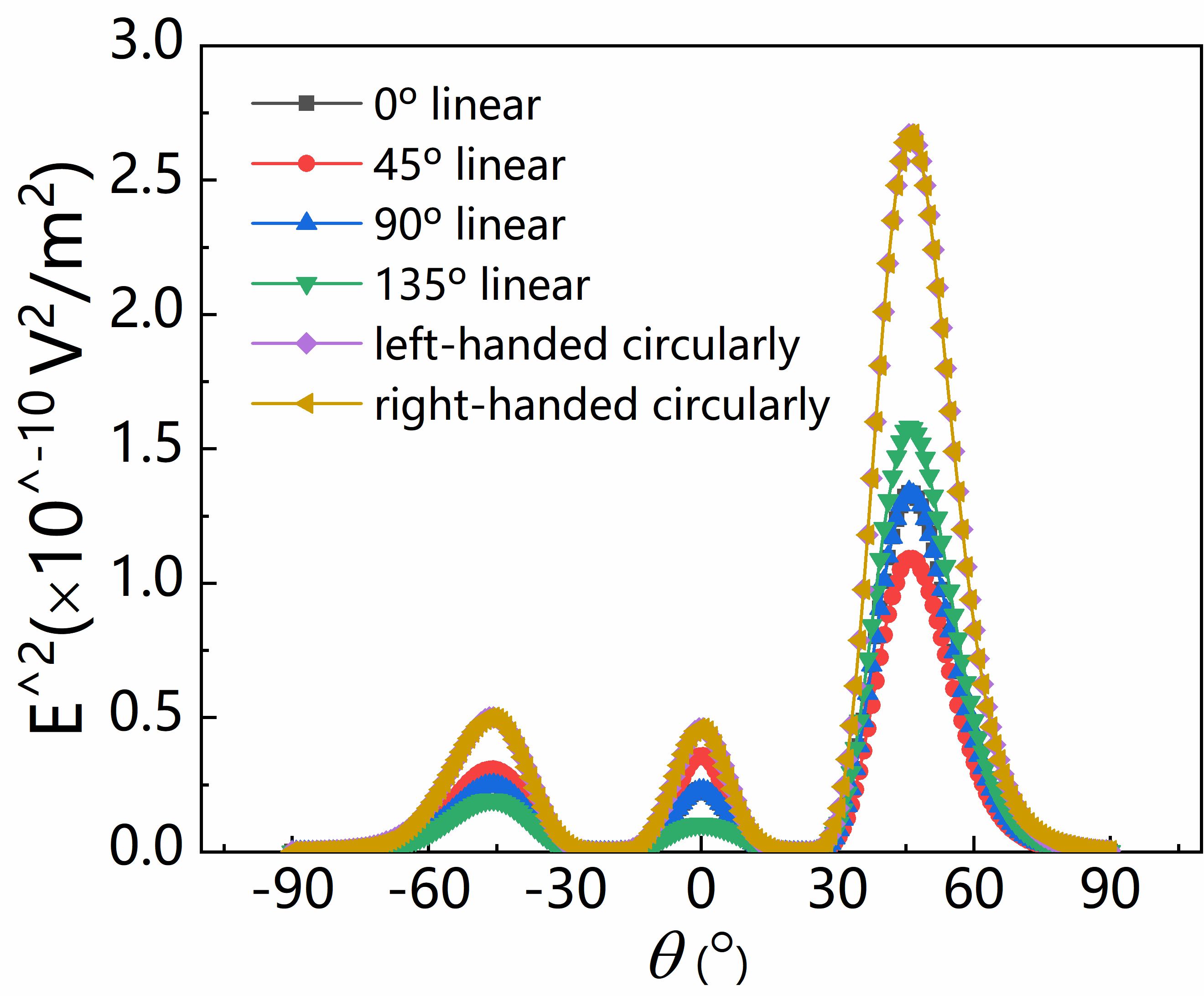


Fig. S4 Far-field electric field intensity as a function of the diffraction zenith angle under different polarized incident light when the diffraction azimuth angle *φ* = 45º, A = 5 and B = 5, (*m*,*n*) = (1,1), the incident wavelength is 1300 nm, and the nanopillars have rotation angles.


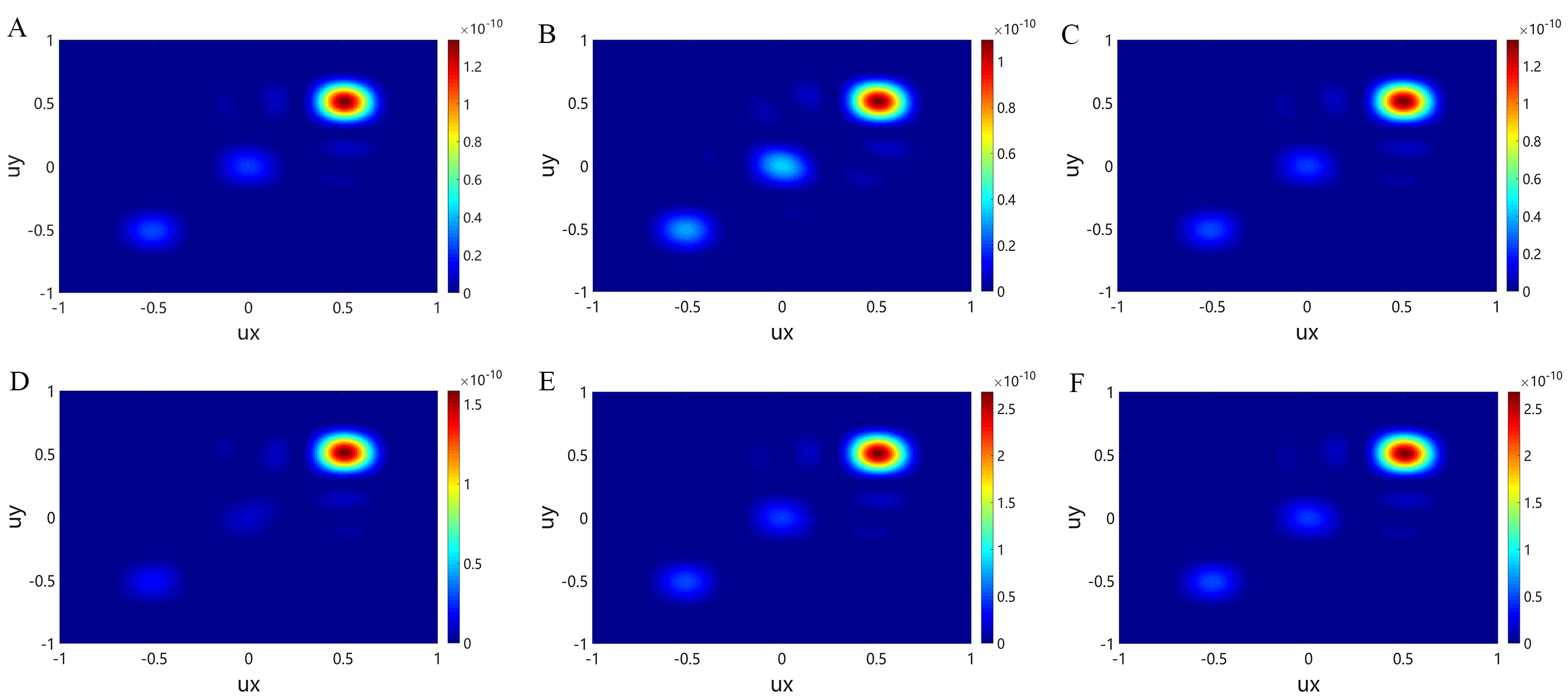


Fig. S5 Far field electric field distribution under different polarized incident light when A = 5 and B = 5, (*m*,*n*) = (1,1), the incident wavelength is 1300 nm, and the nanopillars have rotation angles**.**

(A) 0º linear polarized incident light. (B) 45º linear polarized incident light. (C) 90º linear polarized incident light. (D) 135º linear polarized incident light. (E) Left-handed circularly polarized incident light. (F) Right-handed circularly polarized incident light.


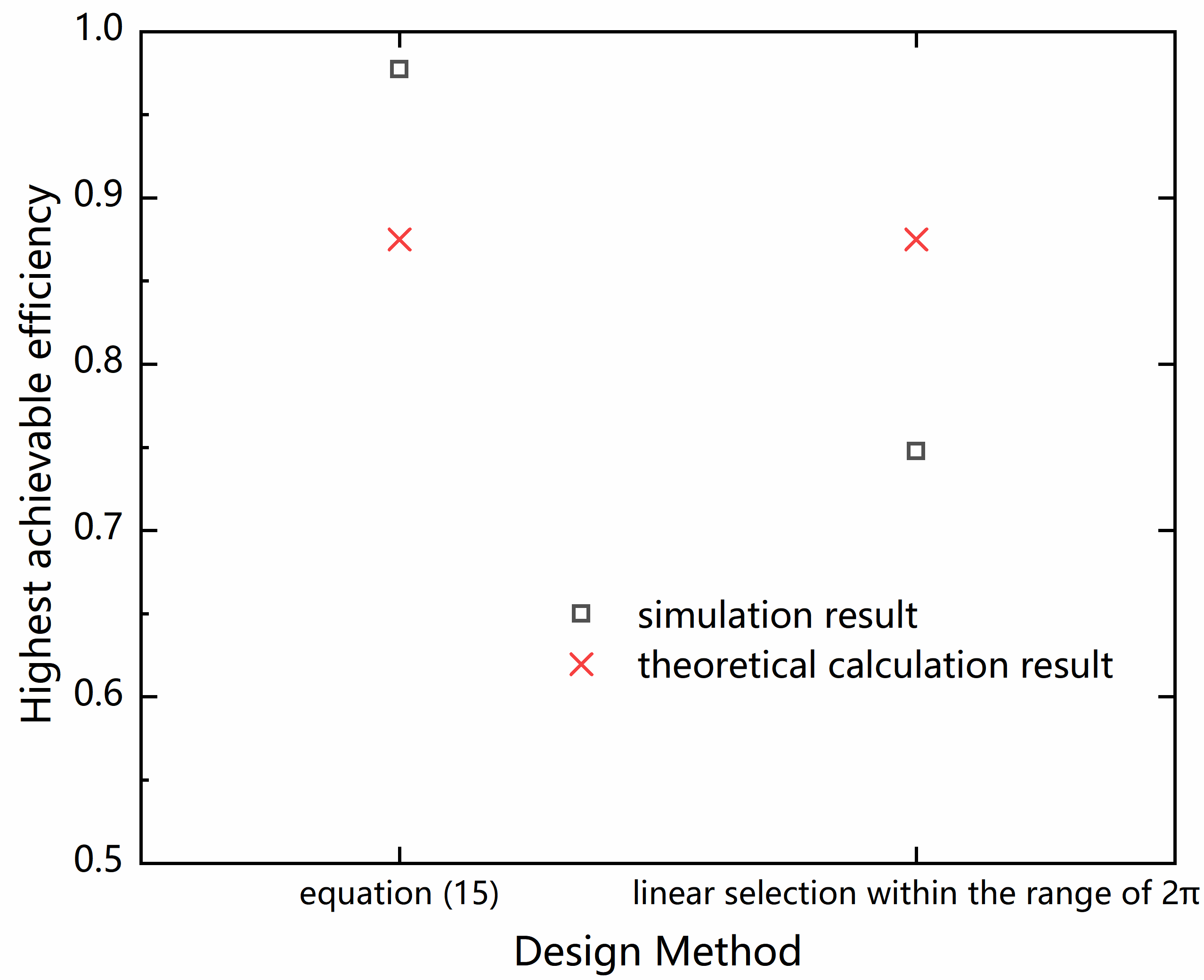


Fig. S6 Comparison between the anomalous refraction efficiency of the incident light transmitted to the (0,1)^th^ order through the two metasurfaces designed by different methods obtained by FDTD simulations and the theoretically achievable maximum efficiency obtained according to Eq. (15) when A = 1 and B = 5, (*m*,*n*) = (0,1), and the incident light is 1300 nm 0º linear polarized light.


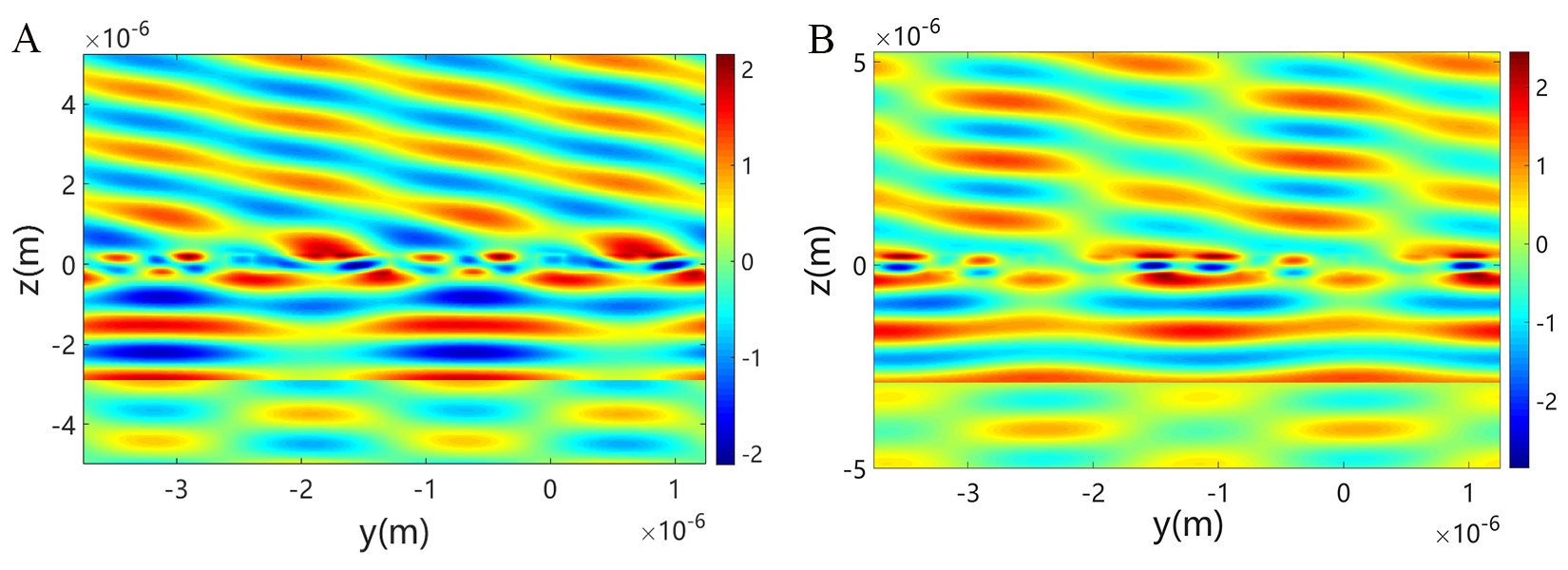


Fig. S7 Ex component of the light transmitted through the two metasurfaces designed by different methods in the yz plane under the incidence of 0º linear polarized light when A = 1 and B = 5, (*m*,*n*) = (0,1), the incident wavelength is 1300 nm, and the nanopillars have no rotation angles.

(A) Designing the metasurface based Eq. (15). (B) Designing the metasurface based on the phase values linearly selected from 0–2π.


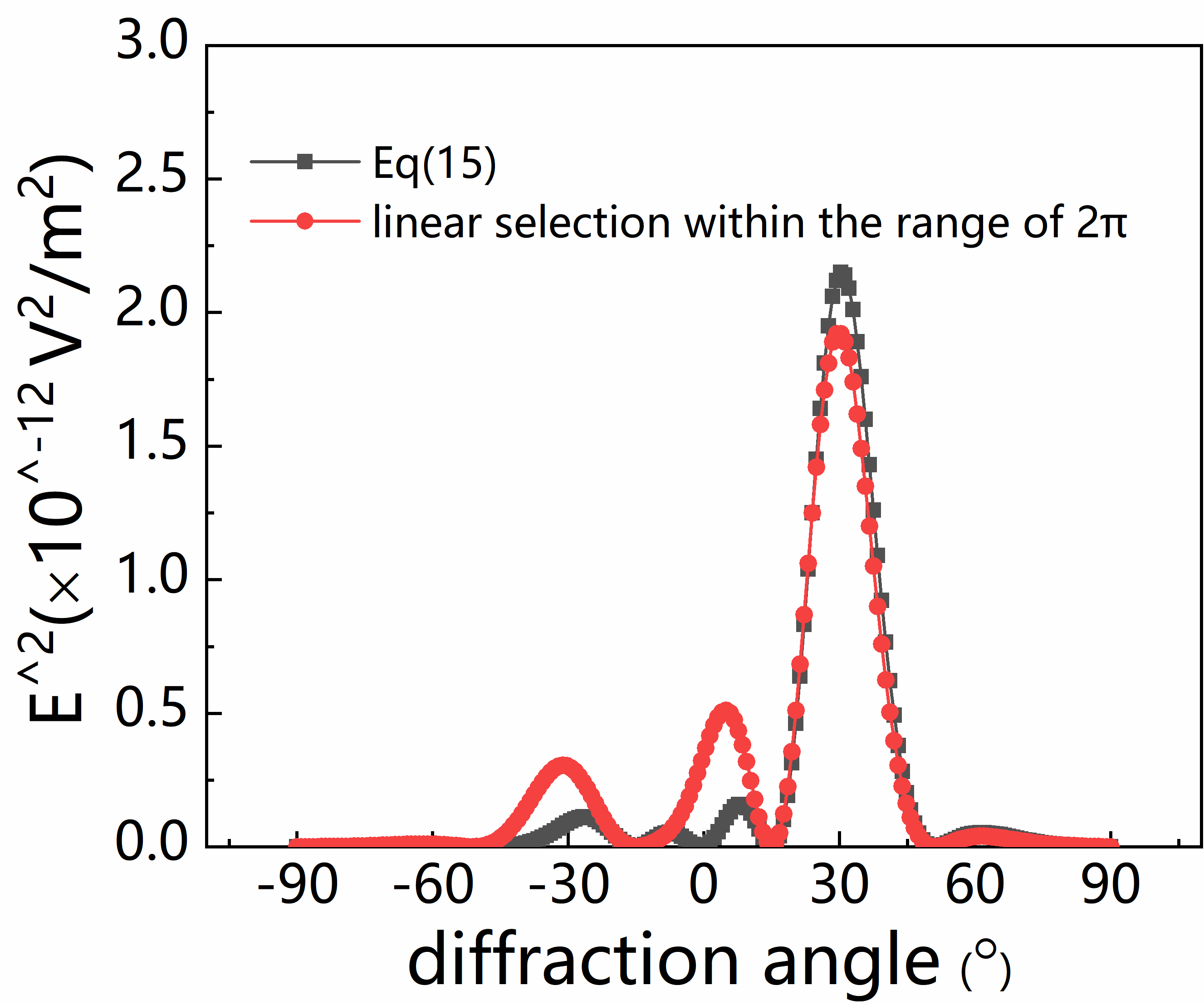


Fig. S8 Far-field electric field intensity of the light transmitted to the (0,1)^th^ order through the two metasurfaces designed by different methods as a function of the diffraction zenith angle under the incidence of 0º linear polarized light when A = 1 and B = 5, (*m*,*n*) = (0,1), the incident wavelength is 1300 nm, and the nanopillars have no rotation angles.


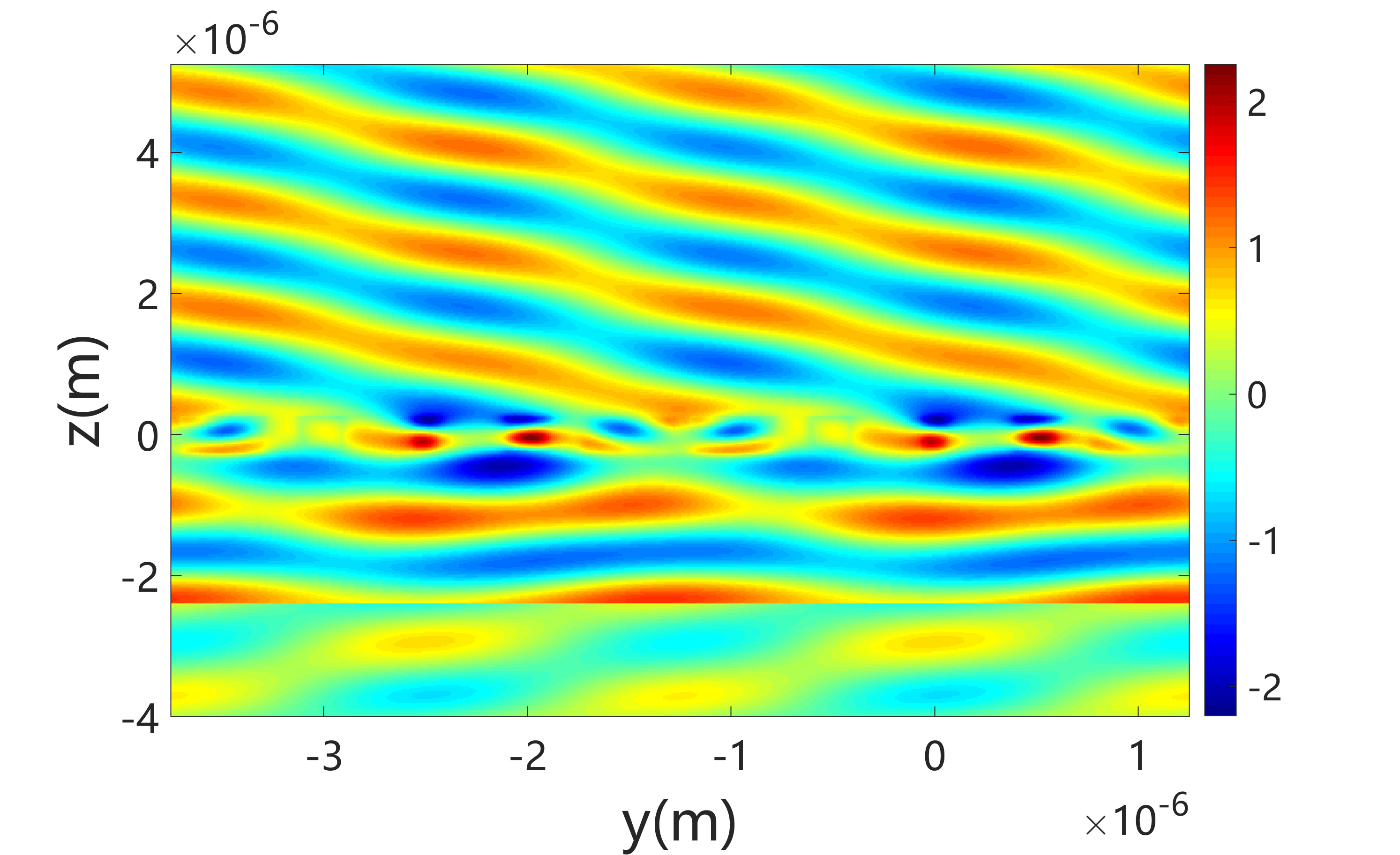


Fig. S9 Ex component of the transmitted light in the yz plane under the incidence of 0º linear polarized light when A = 1 and B = 5, (*m*,*n*) = (0,1), the incident wavelength is 1300 nm, and the nanopillars have rotation angles.


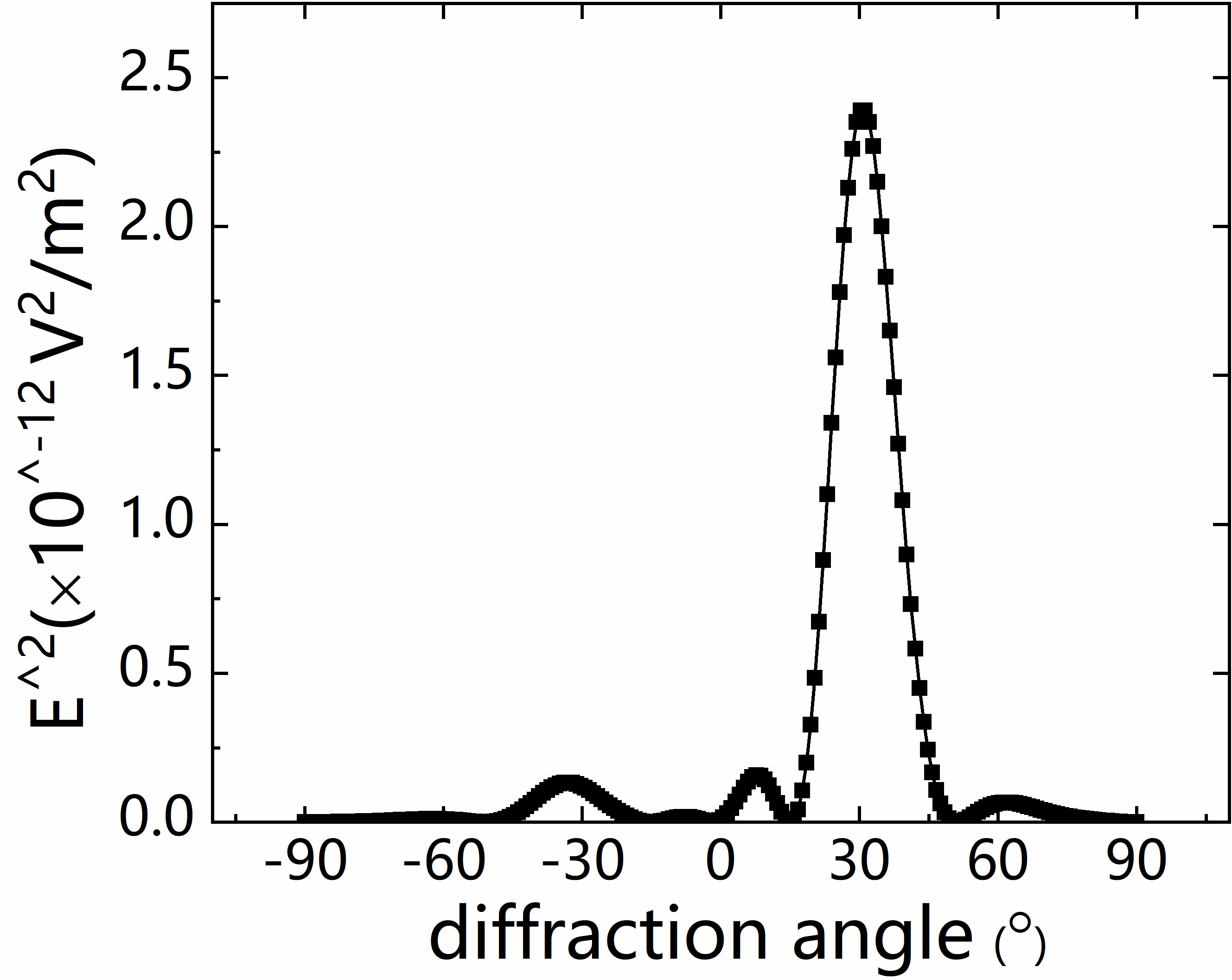


Fig. S10 Far-field electric field intensity as a function of the diffraction zenith angle under the incidence of 0º linear polarized light when A = 1 and B = 5, (*m*,*n*) = (0,1), the incident wavelength is 1300 nm, and the nanopillars have rotation angles.


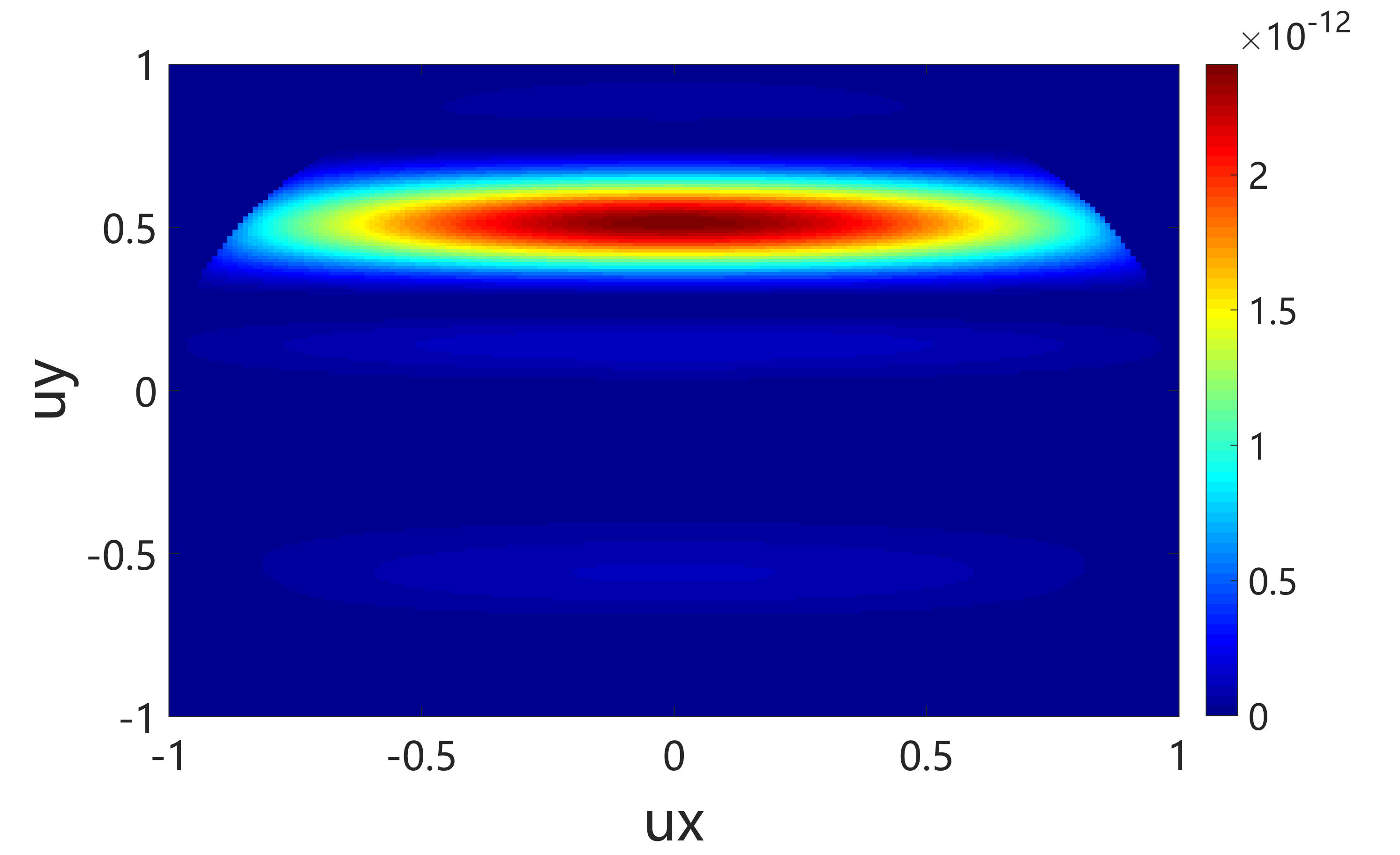


Fig. S11 Far field electric field distribution when A = 1 and B = 5, (*m*,*n*) = (0,1), the incident light is 1300 nm 0º linear polarized light, and the nanopillars have rotation angles**.**


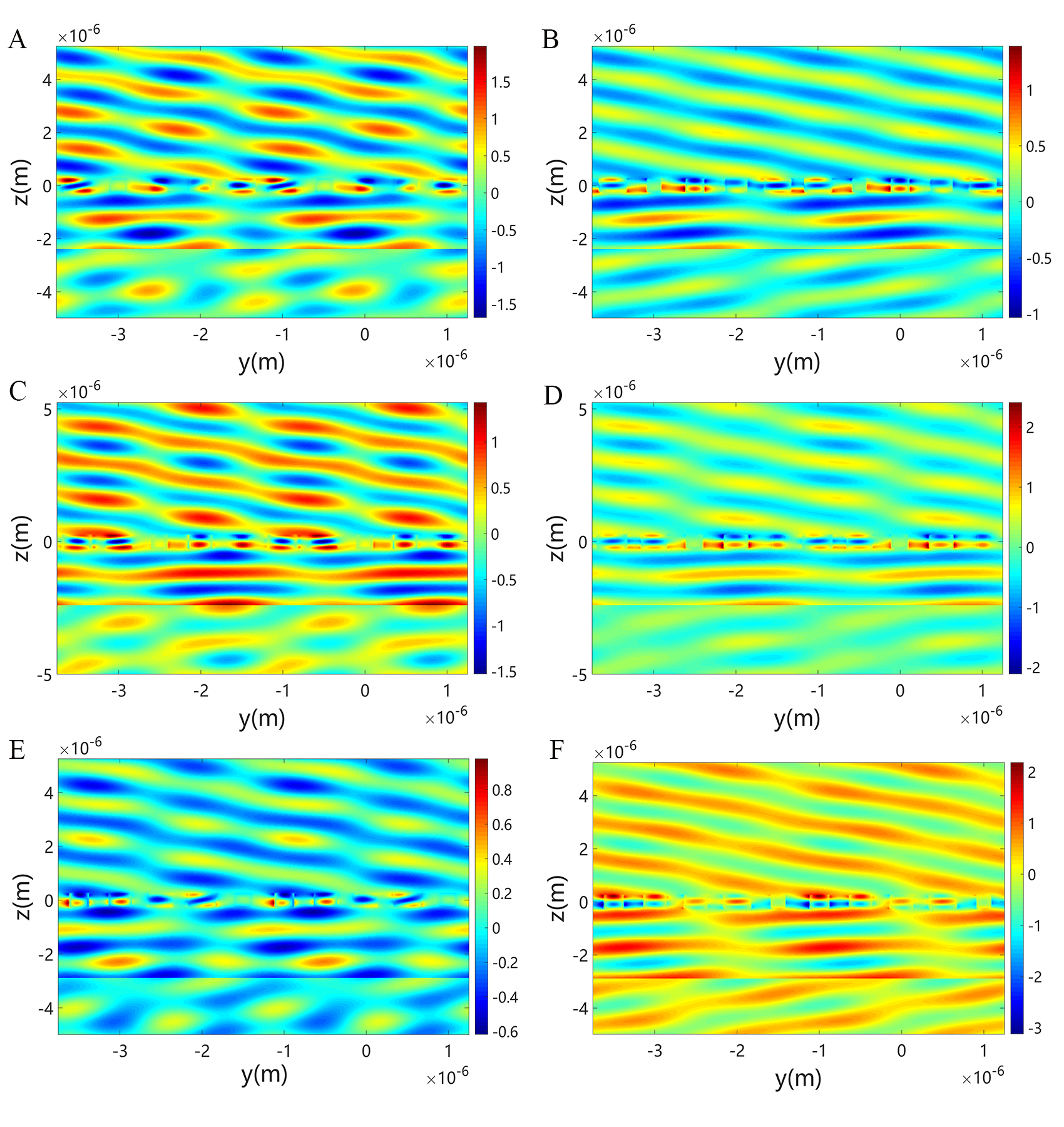


Fig. S12 Corresponding electric field component field patterns in the yz plane under the incidence of 30º, 45º, 108º linear polarized light when A = 1 and B = 5, (*m*,*n*) = (0,1), the incident wavelength is 1200 nm, and the nanopillars have rotation angles.

(A) Ex component of the transmission wave in the yz plane under the incidence of 30º linear polarized light. (B) Ey component of the transmission wave in the yz plane under the incidence of 30º linear polarized light. (C) Ex component of the transmission wave in the yz plane under the incidence of 45º linear polarized light. (D) Ey component of the transmission wave in the yz plane under the incidence of 45º linear polarized light. (E) Ex component of the transmission wave in the yz plane under the incidence of 108º linear polarized light. (F) Ey component of the transmission wave in the yz plane under the incidence of 108º linear polarized light.


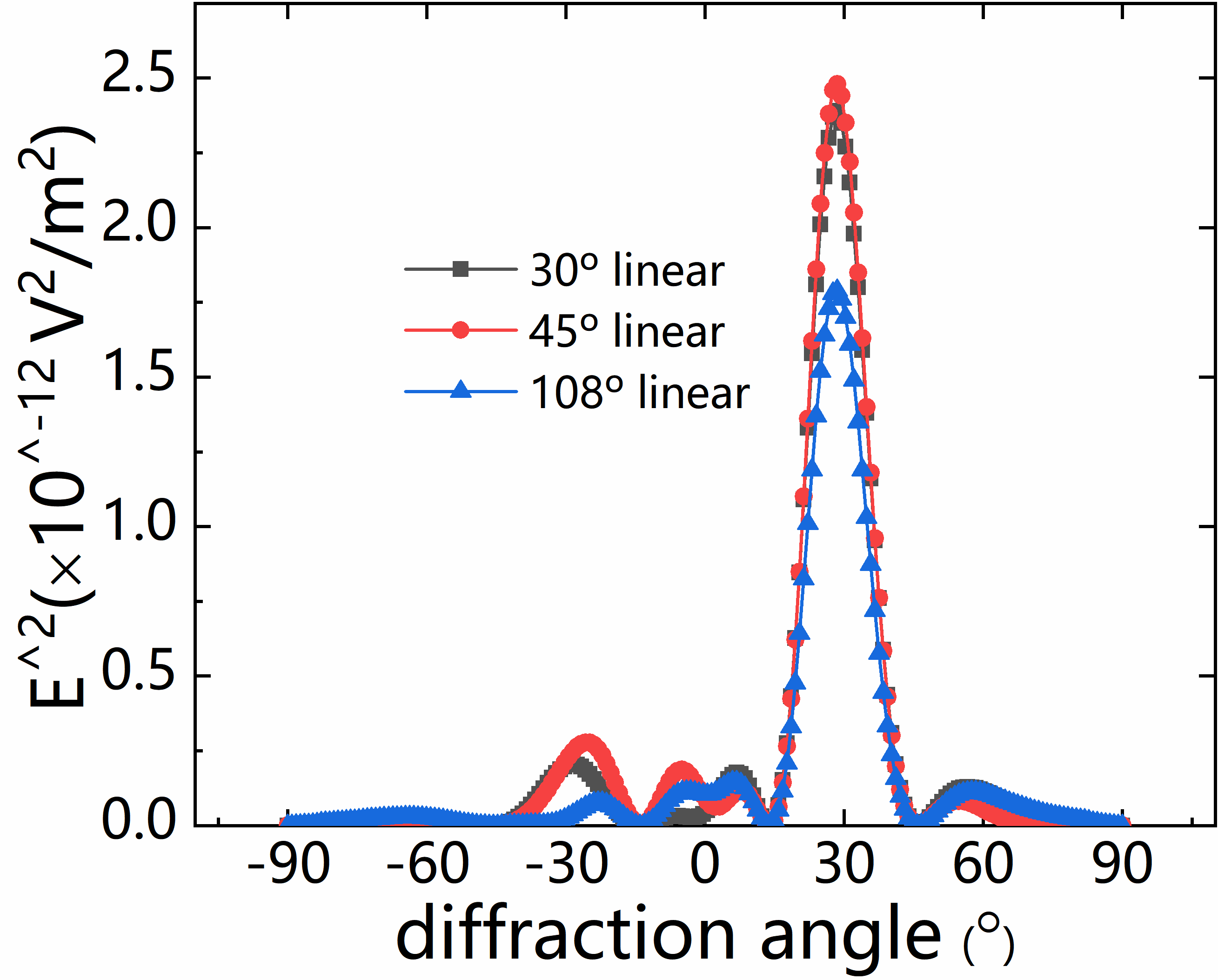


Fig. S13 Far-field electric field intensity as a function of the diffraction zenith angle under the incidence of 30º, 45º, 108º linear polarized light when A = 1 and B = 5, (*m*,*n*) = (0,1), the incident wavelength is 1200 nm, and the nanopillars have rotation angles.


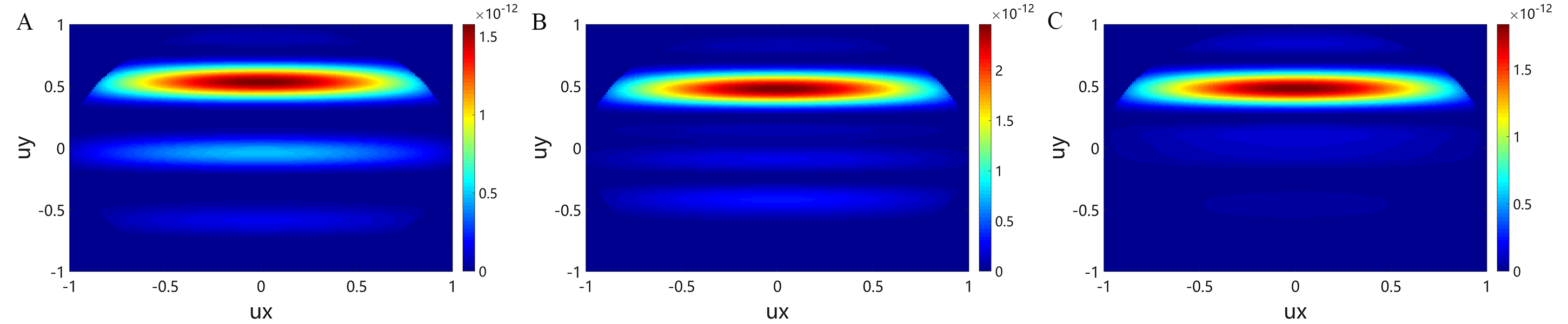


Fig. S14 Far field electric field distribution under the incidence of 30º, 45º, 108º linear polarized light when A = 1 and B = 5, (*m*,*n*) = (0,1), the incident wavelength is 1200 nm, and the nanopillars have rotation angles**.**

(A) Under the incidence of 30º linear polarized light. (B) Under the incidence of 45º linear polarized light. (C) Under the incidence of 108º linear polarized light.


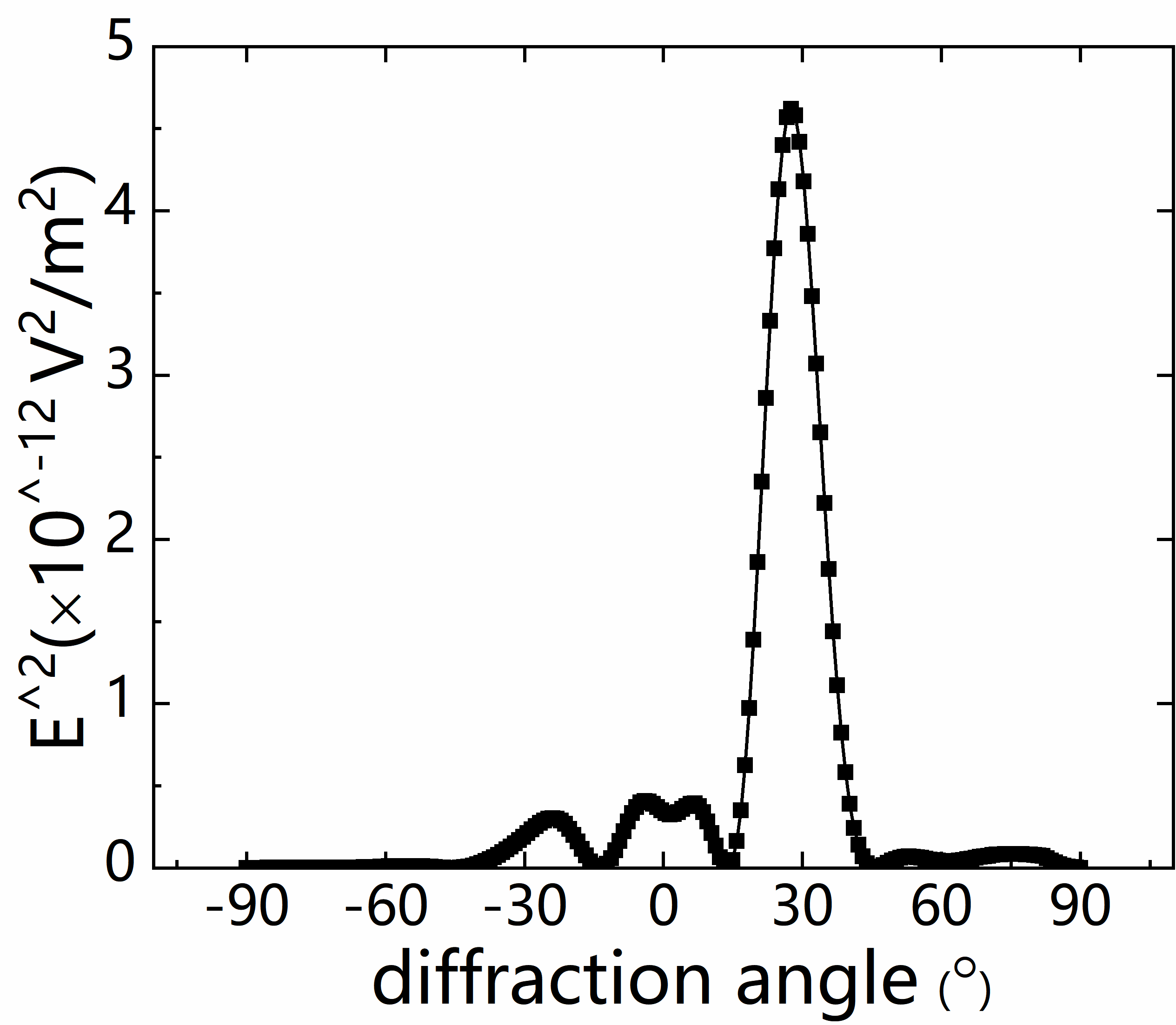


Fig. S15 Far-field electric field intensity as a function of the diffraction zenith angle under the left-handed circularly polarized incident light when A = 1 and B = 5, (*m*,*n*) = (0,1), the incident wavelength is 1190 nm, and the nanopillars have rotation angles.


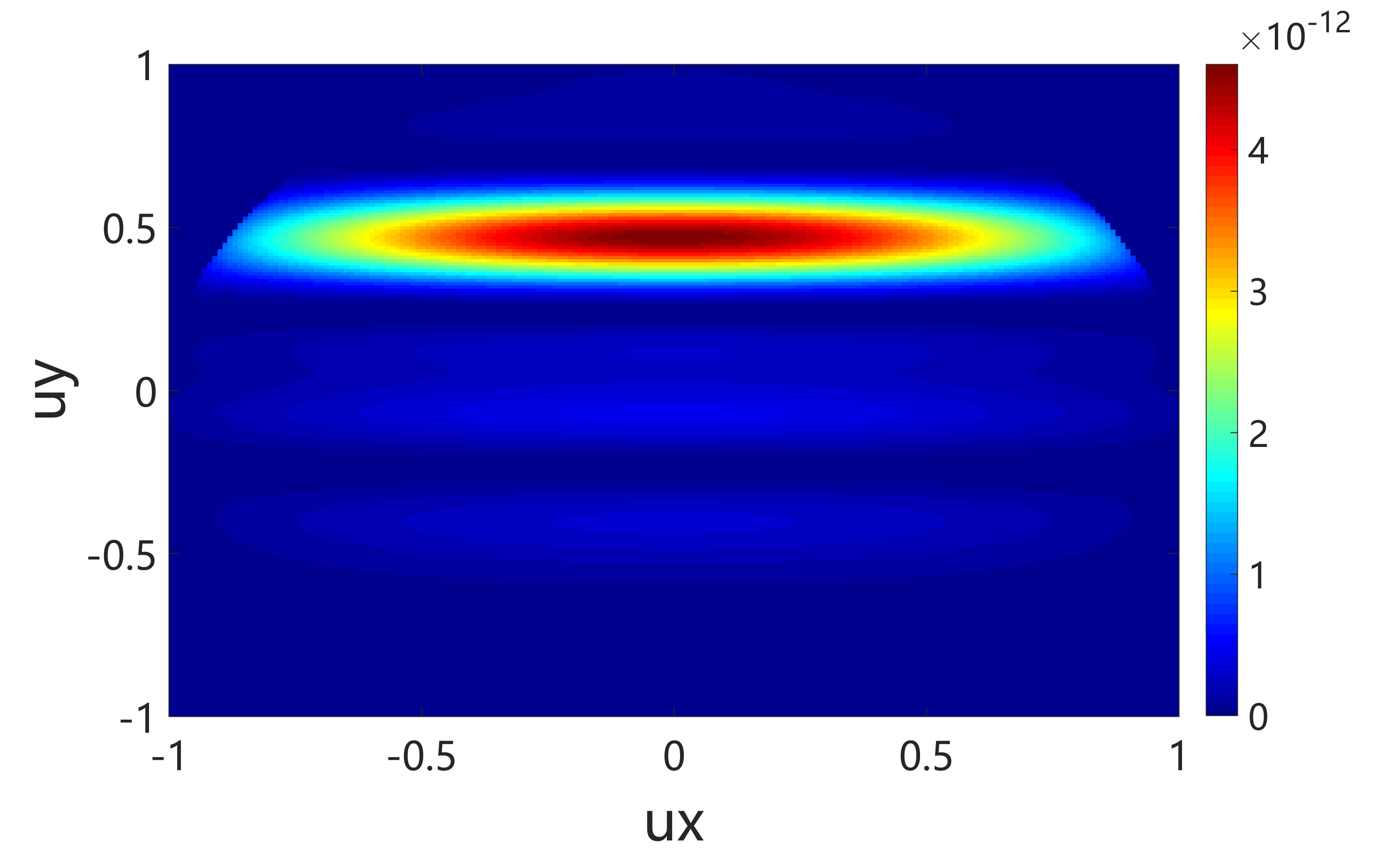


Fig. S16 Far field electric field distribution under the left-handed circularly polarized incident light when A = 1 and B = 5, (*m*,*n*) = (0,1), the incident wavelength is 1190 nm, and the nanopillars have rotation angles.


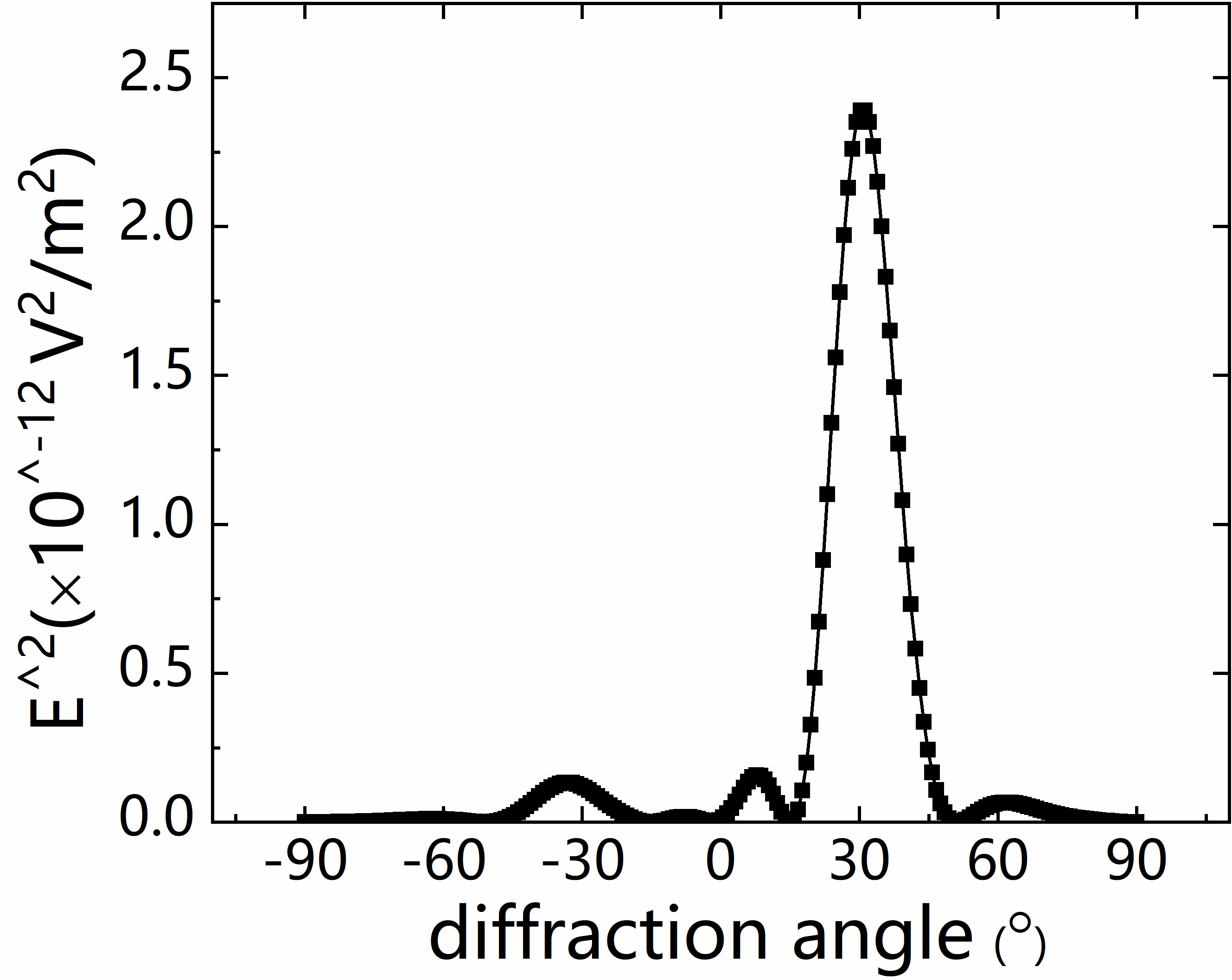


Fig. S17 Far-field electric field intensity as a function of the diffraction zenith angle under the left-handed circularly polarized incident light when A = 1 and B = 5, (*m*,*n*) = (0,1), the incident wavelength is 1200 nm, and when the size of the nanopillars of the metasurface is unchanged and only the rotation angles are changed.


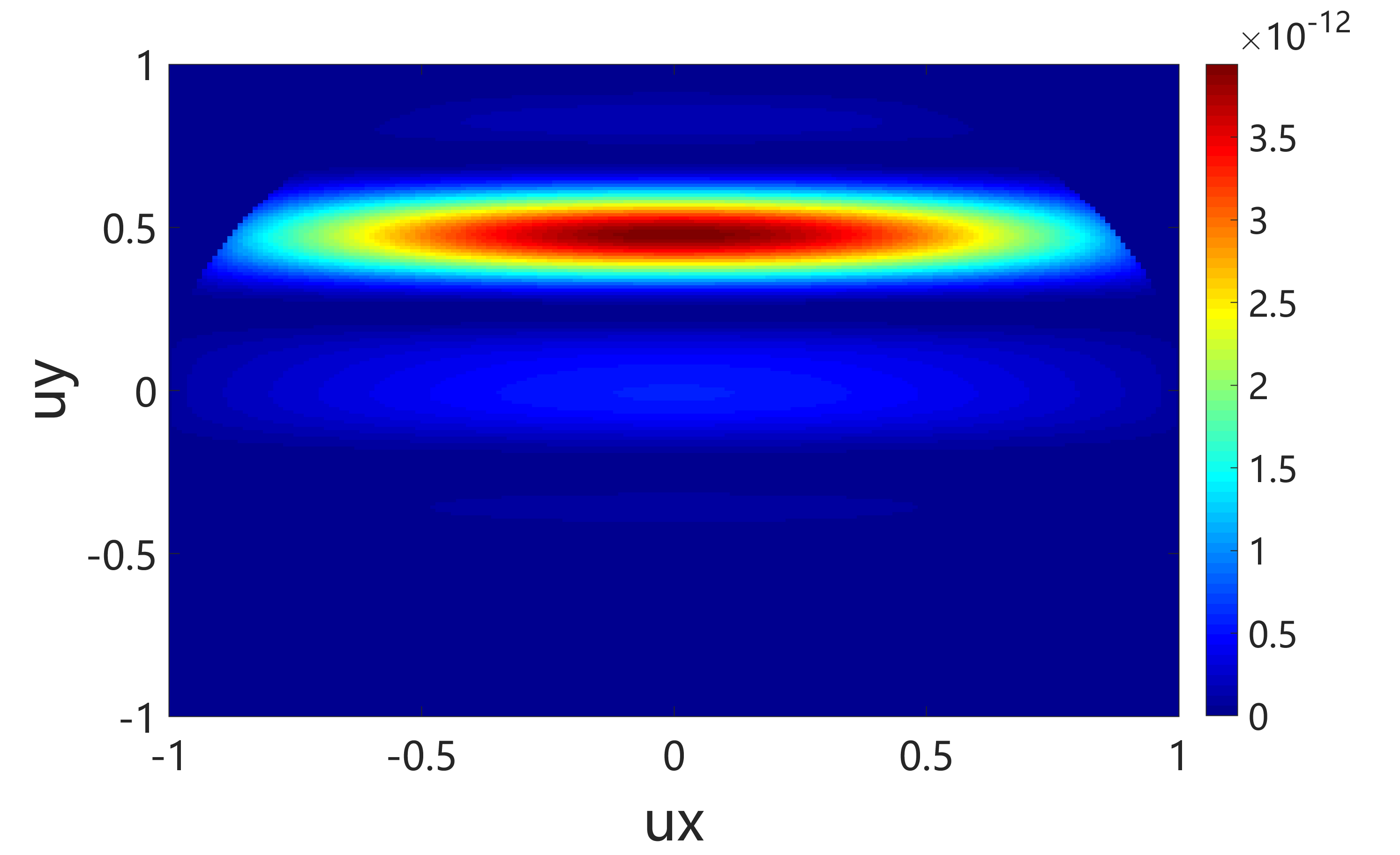


Fig. S18 Far field electric field distribution under the left-handed circularly polarized incident light when A = 1 and B = 5, (*m*,*n*) = (0,1), the incident wavelength is 1200 nm, and when the size of the nanopillars of the metasurface is unchanged and only the rotation angles are changed.


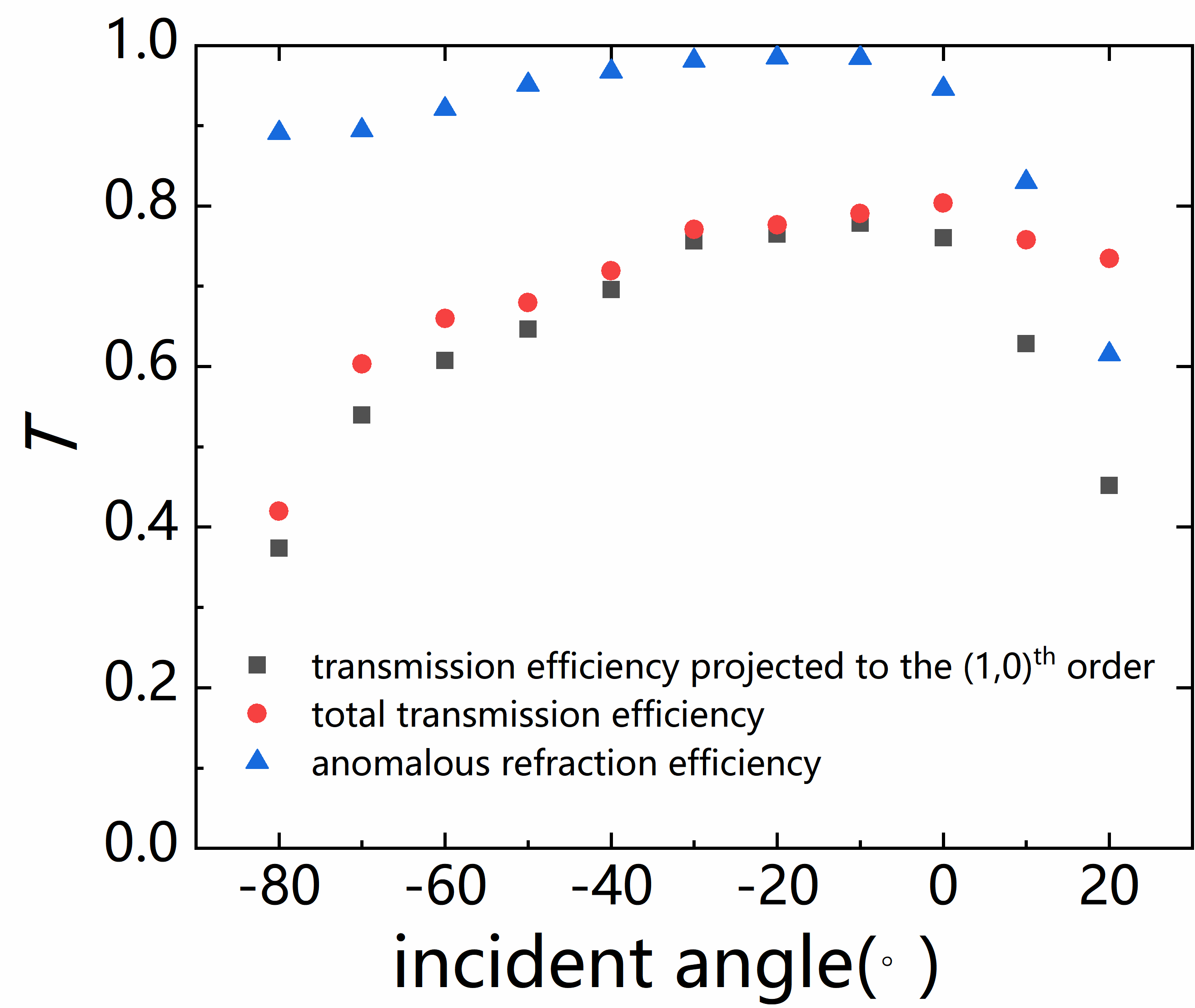


Fig. S19 The simulation results of the total transmission efficiency, the transmission efficiency projected to the (1,0)^th^ order, and the efficiency of anomalous refraction to the (1,0)^th^ order of light passing through the metasurfaces composed by the selected nanopillars under 90º linear polarized light with different incident angle by FDTD when A = 5 and B = 1, (*m*,*n*) = (1,0), the incident wavelength is 1300 nm, and the nanopillars have rotation angles.


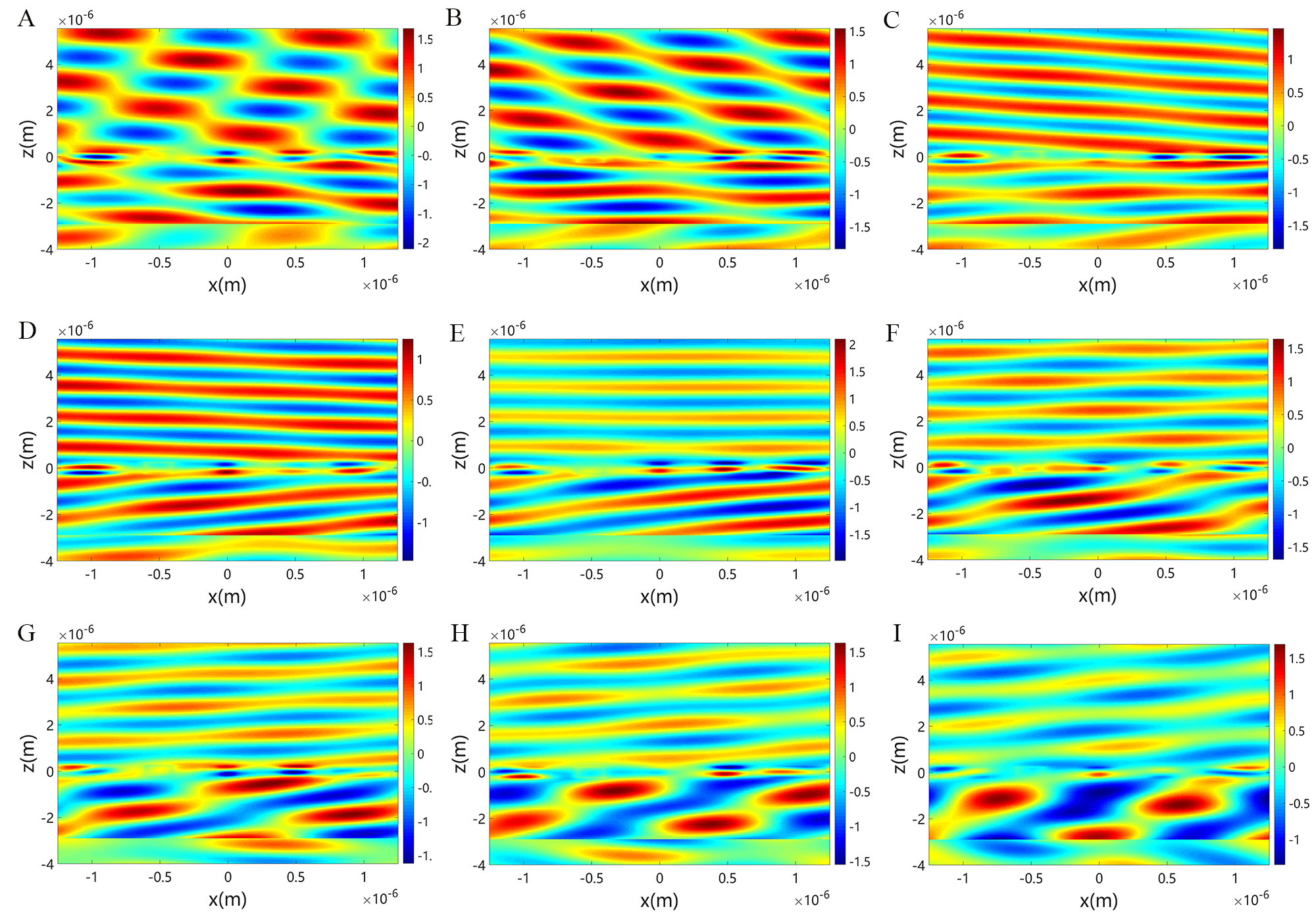


Fig. S20 Ey component of the transmitted light in the xz plane under the incidence of 90º linear polarized light with different incident angle when A = 5 and B = 1, (*m*,*n*) = (1,0), the incident wavelength is 1300 nm, and the nanopillars have rotation angles.

(A) With the incident angle of 20º. (B) With the incident angle of 10º. (C) With the incident angle of -10º. (D) With the incident angle of -20º. (E) With the incident angle of -30º. (F) With the incident angle of -40º. (G) With the incident angle of -50º. (H) With the incident angle of -60º. (I) With the incident angle of -70º.


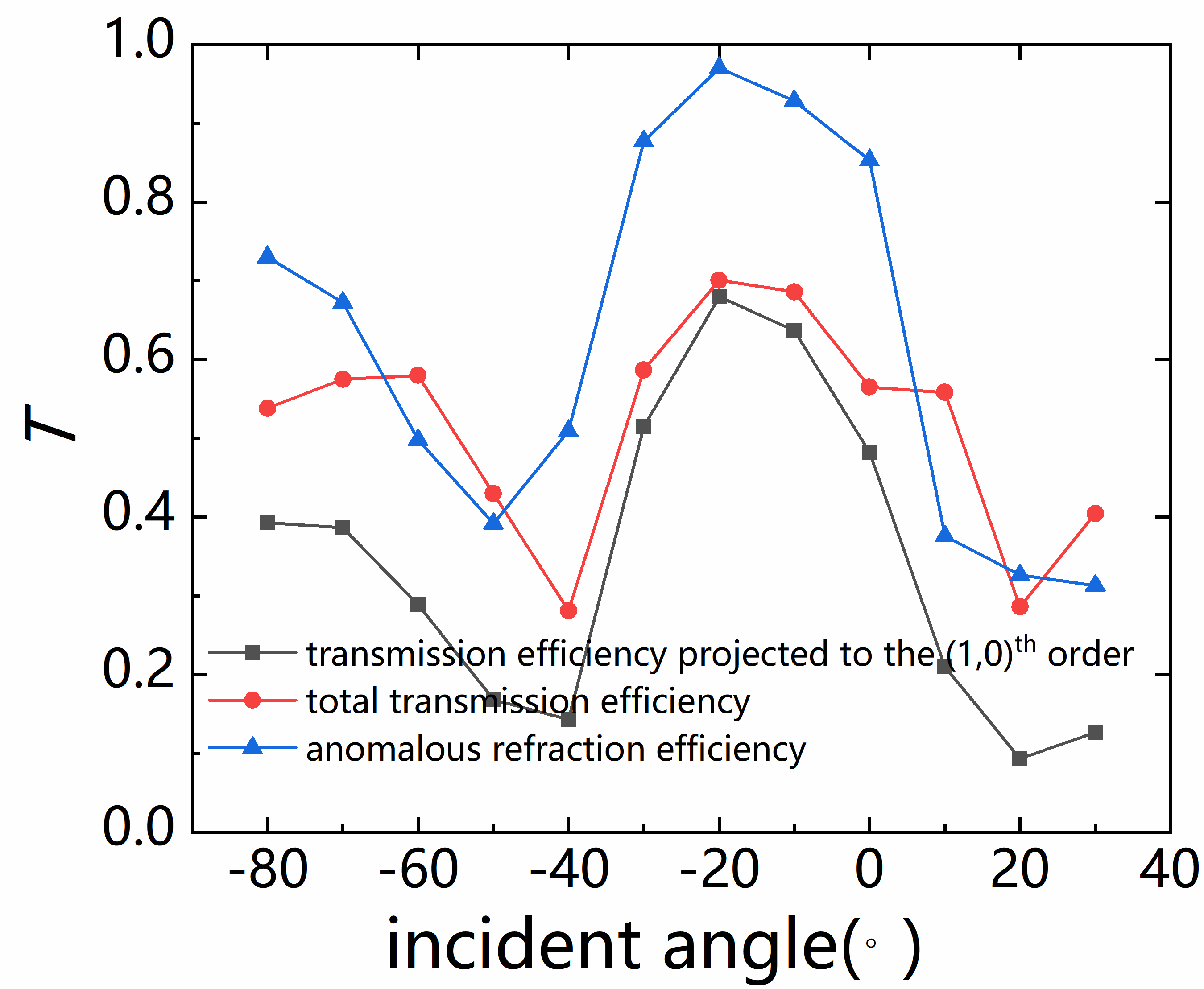


Fig. S21 The simulation results of the total transmission efficiency, the transmission efficiency projected to the (1,0)^th^ order, and the efficiency of anomalous refraction to the (1,0)^th^ order of light passing through the metasurfaces composed by the selected nanopillars under 198º linear polarized light with different incident angle by FDTD when A = 5 and B = 1, (*m*,*n*) = (1,0), the incident wavelength is 1200 nm, and the nanopillars have rotation angles.


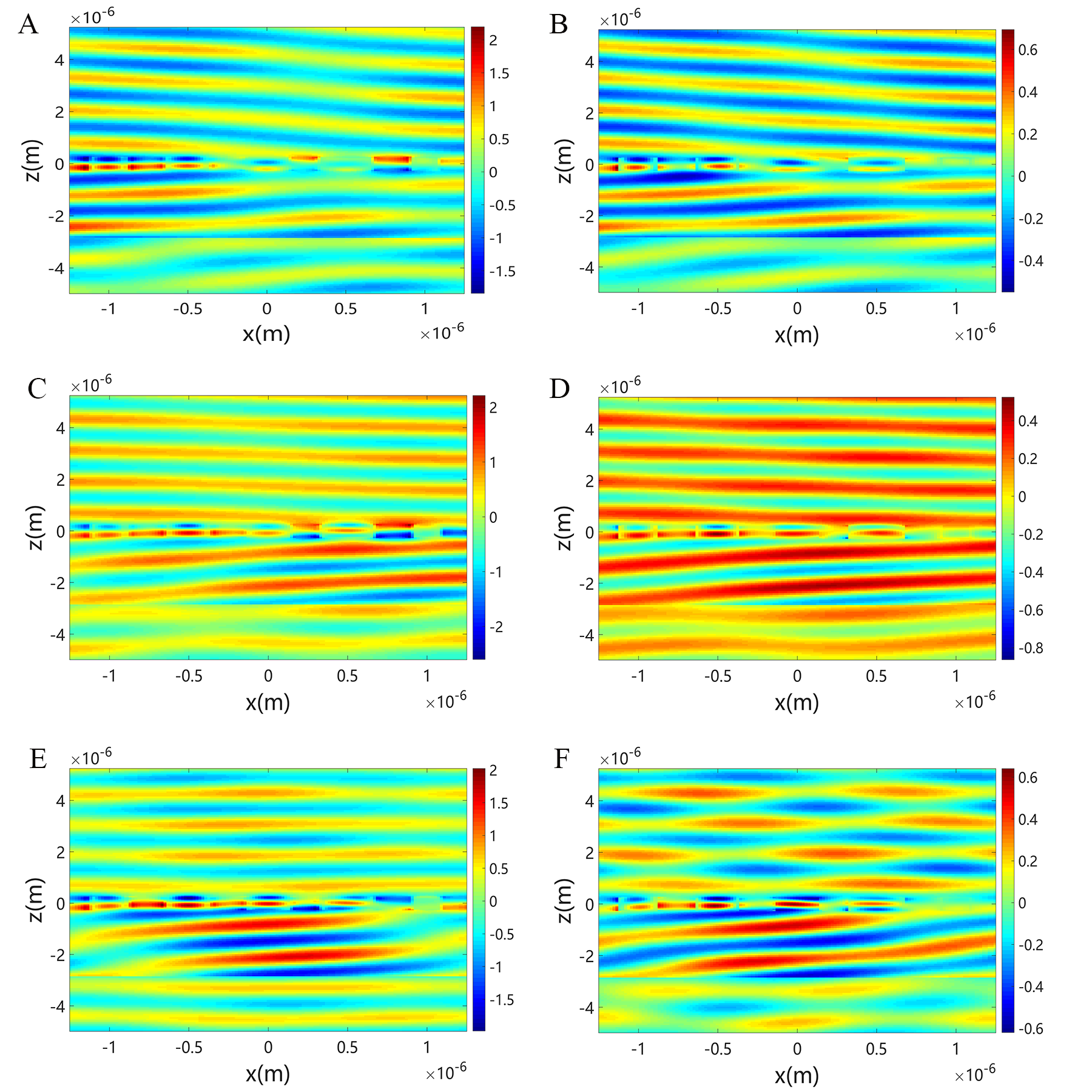


Fig. S22 Electric field component of the transmitted light in the xz plane under the incidence of 198º linear polarized light with different incident angle when A = 5 and B = 1, (*m*,*n*) = (1,0), the incident wavelength is 1200 nm, and the nanopillars have rotation angles.

(A) Ex component of the transmitted light in the xz plane under the incident angle of -10º. (B) Ey component of the transmitted light in the xz plane under the incident angle of -10º. (C) Ex component of the transmitted light in the xz plane under the incident angle of -20º. (D) Ey component of the transmitted light in the xz plane under the incident angle of -20º. (E) Ex component of the transmitted light in the xz plane under the incident angle of -30º. (F) Ey component of the transmitted light in the xz plane under the incident angle of -30º.
